# Oxidative pentose phosphate pathway and glucose anaplerosis support maintenance of mitochondrial NADPH pool under mitochondrial oxidative stress

**DOI:** 10.1002/btm2.10184

**Published:** 2020-09-08

**Authors:** Sun Jin Moon, Wentao Dong, Gregory N. Stephanopoulos, Hadley D. Sikes

**Affiliations:** ^1^ Department of Chemical Engineering Massachusetts Institute of Technology Cambridge Massachusetts USA

**Keywords:** hydrogen peroxide, mitochondria, NADPH, NADPH metabolism, NADPH sensor, oxidative stress, redox kinetic model

## Abstract

Mitochondrial NADPH protects cells against mitochondrial oxidative stress by serving as an electron donor to antioxidant defense systems. However, due to technical challenges, it still remains unknown as to the pool size of mitochondrial NADPH, its dynamics, and NADPH/NADP^+^ ratio. Here, we have systemically modulated production rates of H_2_O_2_ in mitochondria and assessed mitochondrial NADPH metabolism using iNap sensors, ^13^C glucose isotopic tracers, and a mathematical model. Using sensors, we observed decreases in mitochondrial NADPH caused by excessive generation of mitochondrial H_2_O_2_, whereas the cytosolic NADPH was maintained upon perturbation. We further quantified the extent of mitochondrial NADPH/NADP^+^ based on the mathematical analysis. Utilizing ^13^C glucose isotopic tracers, we found increased activity in the pentose phosphate pathway (PPP) accompanied small decreases in the mitochondrial NADPH pool, whereas larger decreases induced both PPP activity and glucose anaplerosis. Thus, our integrative and quantitative approach provides insight into mitochondrial NADPH metabolism during mitochondrial oxidative stress.

## 1 INTRODUCTION

Mitochondria emerge as a major source of reactive oxygen species (ROS), and excessive production of ROS has been linked to various diseases including neurodegeneration, inflammation, aging, diabetes, and cancers via induction of lipid peroxidation, protein oxidation, and DNA damage.^1^, ^2^, ^3^, ^4^, ^5^, ^6^, ^7^ Of various types of ROS, hydrogen peroxide (H_2_O_2_) acts as a signaling molecule that can initiate expression of survivor genes such as antioxidant response elements (e.g., Nrf2), induce DNA repair mechanisms (e.g., p53 and ATM), or activate programmed cell death pathways (e.g., NF‐ĸB).^4^, ^8^, ^9^ As accumulation of H_2_O_2_ can induce toxicity, cells maintain a defensive system to clear H_2_O_2_ via redox reactions.^2^, ^10^, ^11^

In the antioxidant network, NADPH plays a critical role by serving as a reductant during removal of H_2_O_2_ to maintain redox homeostasis. It donates two electrons to reduce oxidized cysteine residues of thioredoxin via thioredoxin reductase, or glutathione via glutathione reductase.^12^, ^13^, ^14^, ^15^ Thioredoxin with reduced cysteine residues reacts with peroxiredoxins, which have been known to be the major scavenger of H_2_O_2_ based on their abundance and fast second order rate coefficient compared to glutathione peroxidase reaction at low levels of intracellular H_2_O_2_. ^16^, ^17^ In mitochondria, peroxiredoxin 3 is known to scavenge 90% of H_2_O_2_, suggesting peroxiredoxin‐thioredoxin‐NADPH as the major clearance pathway for mitochondrial H_2_O_2_. ^16^ In parallel, glutathione reacts with glutareredoxin which serves as a reductase for oxidized proteins, and with glutathione peroxidase during direct reactions with H_2_O_2_.^18^, ^19^

Oxidative stress, a condition caused by an inadequate clearance or excessive production of H_2_O_2_, has been reported to decrease the total NADPH pool, NADPH/NADP^+^ ratio, and modulate NADP‐dependent metabolic fluxes.^12^, ^13^, ^20^ For instance, oxidative stress in fibroblast cells led to a shift of glycolytic flux toward the oxidative pentose phosphate pathway to regenerate NADPH.^20^, ^21^, ^22^, ^23^ In isolated cardiac myocytes under a pathological workload, the direction of mitochondrial nicotinamide transhydrogenase reaction was reversed, lowering the total NADPH pool and increasing the production of mitochondrial ROS.^24^ Similarly, the availability of NADPH in mitochondria along with NADPH‐producing enzymes such as isocitrate dehydrogenase 2 (IDH2) and nicotinamide nucleotide transhydrogenase (NNT) was shown to control the antioxidant network for clearance of H_2_O_2_.^25^

To our knowledge, little has been known about the causal relationship between oxidative stress derived within mitochondria and mitochondrial or cytosolic NADPH pools. Previously, the total NADPH level or its ratio to NADP^+^ has been reported to decrease by exogenous oxidative stress introduced extracellularly or to the exterior of isolated mitochondria, but no direct evidence of compartmentalized NADPH dynamics by mitochondria specific oxidative stress in living cells. Gas or liquid chromatography coupled to mass spectrometry and enzymatic cycling assays provide great sensitivity and specificity for measurement of NADPH, but they are based on measurements of the average of cell lysates, making it difficult to preserve spatial and temporal information of NADPH in living cells at single cell resolution.^26^, ^27^

Co‐expressing compartment‐specific NADPH sensors and D‐amino acid oxidase (DAAO) which is used as H_2_O_2_ generator, we evaluated mitochondrial and cytosolic NADPH dynamics upon localized H_2_O_2_stress first time. iNap sensors were genetically‐encoded probes for NADPH and provided a wide dynamic range with a ratiometric fluorescent readout, which could be simply recorded using a fluorescence microscope at single‐cell resolution.^28^ For mitochondrial NADPH experiments, we expressed the sensors using mitochondria using localization tags. Fluorescence ratio was defined as a ratio between fluorescence emissions at 515 nm excited at 415 and 488 nm ($R=\frac{F_{\mathrm{em}:515,\mathrm{ex}:415}}{F_{\mathrm{em}:515\;\mathrm{nm},\mathrm{ex}:488}}$). Due to the influence of pH fluctuations to iNap fluorescence at 488 nm, the fluorescence ratio of iNap could be normalized to that of iNapC, a control iNap sensor engineered to lose its binding affinity to NADPH. As cytoplasmic pH was shown to be stable during oxidative stress,^28^ we used the fluorescence ratio (R) for cytoplasmic iNap experiments. For mitochondrial iNap experiments, we used a normalized form of fluorescence ratio, defined as fluorescence readout ($R^{\prime }=\frac{R_{\text{iNap}-\text{mito}}\;}{R_{\text{iNapC}-\text{mito}}}$). For kinetic experiments, fluorescence ratio or readout were normalized to initial values. For modulation of mitochondrial H_2_O_2_ level, we expressed DAAO with a mitochondrial localization sequence. Upon addition of varying concentrations of D‐alanine, reactions with oxygen produced H_2_O_2_ as a byproduct.^29^

Next, we further investigated whether the activities of NADPH generation pathways were altered in response to changes in NADPH pools caused by increasing mitochondrial H_2_O_2_. Using [1,2‐^13^C_2_]glucose and [U‐^13^C_6_]glucose, we examined the activity of the pentose phosphate pathway (PPP) and labeling patterns of TCA cycle metabolites. The PPP, occurring in cytoplasm, is known as the major site of NADPH production via glucose 6‐phosphate dehydrogenase (G6PD) and 6‐phosphogluoconate dehydrogenase (6GPD), and increased activity of the PPP has been identified as a key characteristic of cancer metabolism.^23^, ^30^ In mitochondria, TCA cycle metabolites such as citrate and malate can be used to regenerate NADPH via IDH2 and malic enzymes (ME3), respectively. Additionally, nicotineamide nucleotide transhydrogenase (NNT) is located in the mitochondrial membrane, maintaining mitochondrial NADPH/NADP^+^ by converting NADP^+^ to NADPH at the expense of NADH.^31^

Lastly, using a mathematical model based on a network of redox reactions, we examined the extent of mitochondrial NADPH/ NADP^+^ during mitochondrial oxidative stress first time. Previous redox models were designed to evaluate cytosolic H_2_O_2_ metabolism such as its diffusion rates across the extracellular membrane and rate parameters with antioxidant substrates.^32^, ^33^, ^34^, ^35^ However, these models were based on the values of cytoplasmic kinetic parameters and the generation rate of NADPH was designated to the G6PD enzyme, which was expressed solely in cytosol.^36^ Mitochondrial NADPH could be regenerated via reactions catalyzed by isoforms of NADP‐dependent enzymes such as ME3, IDH2, glutamate dehydrogenase 1 (GLUD1), methylenetetrahydrofolate 2 (MTHFD2), aldehyde dehydrogenase, and NNT.^37^ Thus, we adapted the model by updating initial concentrations of redox species in mitochondria and kinetic parameter values compiled in a bioinformatics database.^16^, ^38^

## 2 RESULTS

### 2.1 Expression of DAAO and iNap in mitochondria enables mitochondria‐specific production of H_2_O_2_ and measurement of NADPH

**FIGURE 1:**
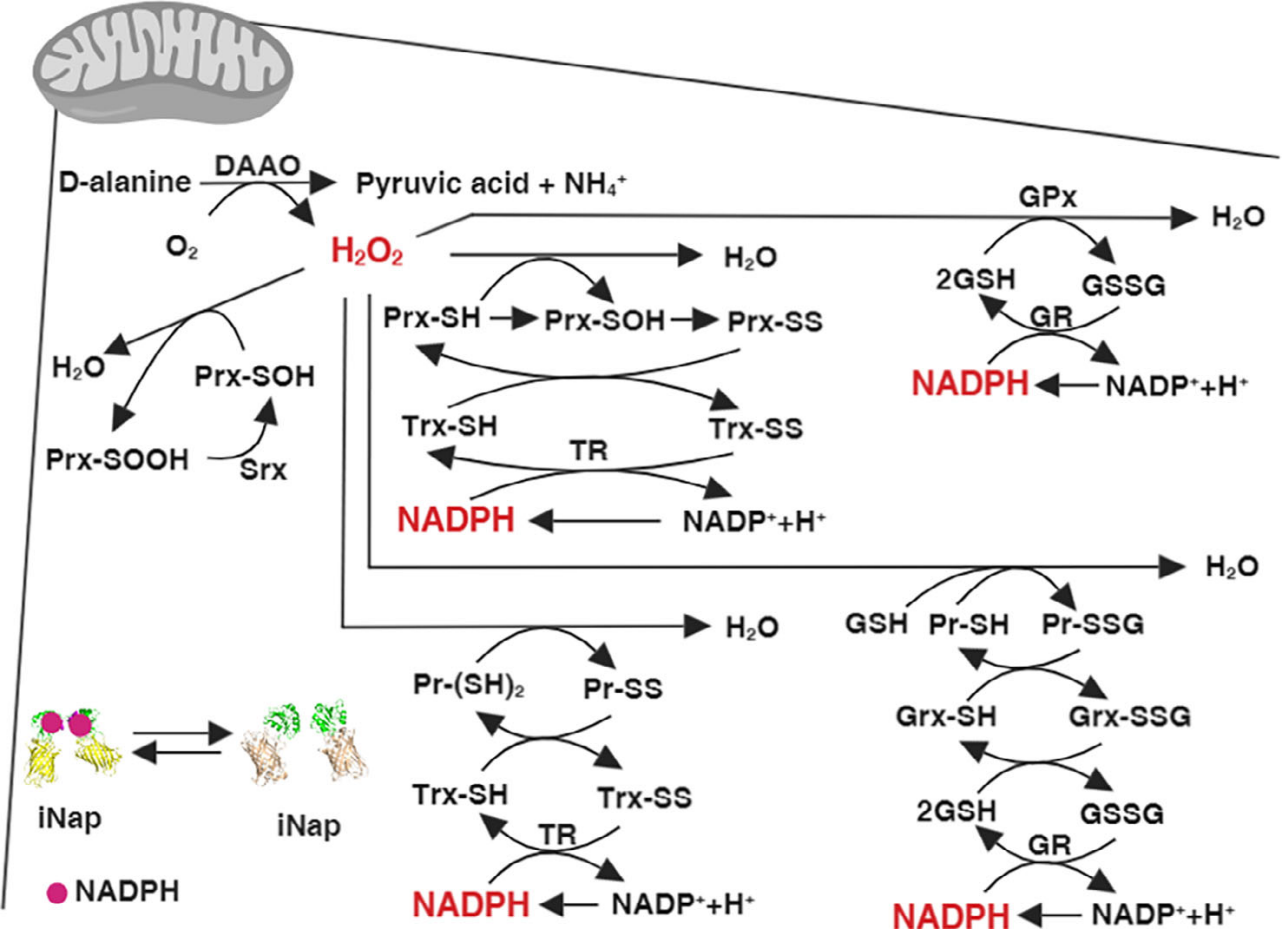
Schematics representing a mechanistic connection between hydrogen peroxide and NADPH via a network of mitochondrial redox reactions. We modulated mitochondrial H_2_O_2_ using D‐amino acid oxidase (DAAO) targeted to mitochondria. H_2_O_2_ is reduced to water by reacting with reactive cysteine residues of Prx, GPx, or proteins. Through subsequent redox cycling reactions, NADPH acts as an ultimate reductant by donating electrons to oxidized redox species. The mitochondrial NADPH pool was monitored by measuring a fluorescence ratio from a mitochondrial iNap sensor, a genetically encoded sensor for NADPH. Detailed mitochondrial redox reactions considered in our system can be found in Table S1

**FIGURE 2:**
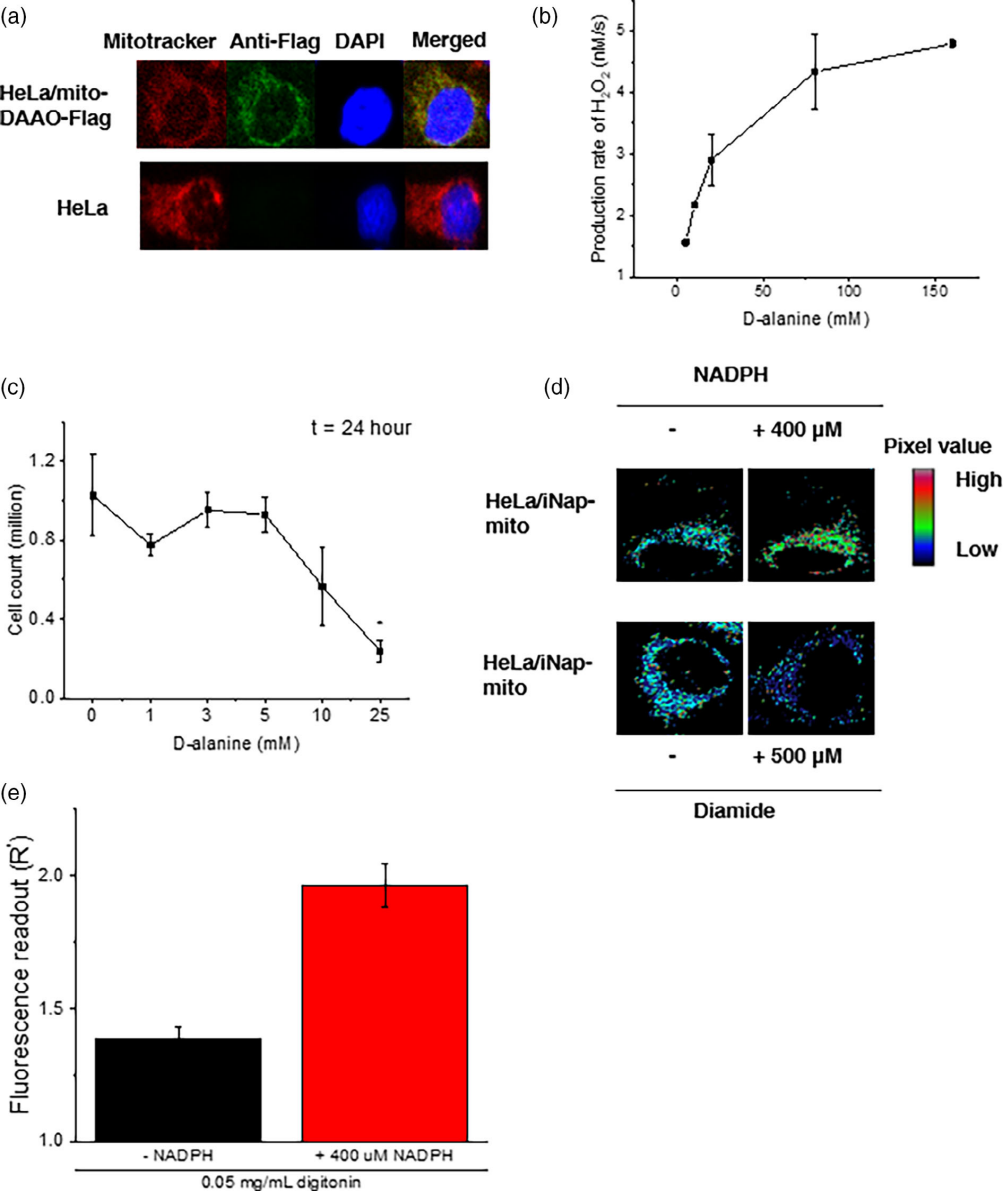
Validation of a system that generates hydrogen peroxide in mitochondria with D‐amino acid oxidase (DAAO) and measures NADPH with an iNap sensor. (a) Hela cells were transiently transfected with a mito‐DAAO‐FLAG and its localization to mitochondria was confirmed. Staining: Mitotracker (red), anti‐FLAG (green), DAPI (blue). (b) The enzymatic activity of DAAO was measured via a horseradish peroxidase based Amplex UltraRed assay. Fluorescence intensity was measured after incubation of HeLa cell lysates with D‐alanine for an hour. Fluorescence readings were converted to hydrogen peroxide concentrations using a standard curve constructed using known concentrations of hydrogen peroxide. Data represent two independent experiments ±*SD*. (c) Cell numbers were counted after 24 hr of incubation of D‐alanine with Hela/mito‐DAAO cells with error bars representing SEM of three independent experiments. (d) Pseudo‐colored images represent the change of fluorescence intensity of Hela/mito‐iNap cells in the presence or absence of NADPH, or diamide. For incubation of NADPH, 0.05 mg/ml digitonin was used to permeabilize the mitochondrial membrane. (e) Fluorescence readout ($R^{\prime }=\frac{R_{\text{iNap}-\text{mito}}\;}{R_{\text{iNapC}-\text{mito}}})$ was quantified before and after addition of 400 μM NADPH in digitonin treated Hela cells expressing iNap sensors. Data represents the mean of fluorescence ratio of individual cells from three independent experiments ±*SEM*. (*n* = 22 and 16 cells)

We designed a system capable of producing H_2_O_2_ and measuring NADPH simultaneously (Figure 1). First, a plasmid that encodes DAAO enzyme was constructed with a mitochondrial localization sequence at the N terminus and FLAG tag at the C terminus. The localization was confirmed via immunofluorescence staining with anti‐Flag antibody, Mitotracker, and DAPI in fixed Hela cells that transiently expressed the mito‐DAAO construct (Figures 2a, S1A–D). Next, enzymatic activity was tested using a horseradish peroxidase based Amplex UltraRed assay in response to varying concentrations of D‐alanine added to lysed Hela cells with mito‐DAAO (Figure 2b). Using the Michaelis–Menten kinetic relation and the previously determined turnover rate of DAAO enzyme, we calculated DAAO concentration in mitochondria to be ~0.6 μM per cell, assuming the radius of a Hela cell is 10 μm and the mitochondrial volume comprises 10% of the total volume.^39^ A maximum peroxide generation rate, v_*max*_, was estimated to be 2.0 × 10^−4^ M/s per cell, and the K_*m*_ to be 10.4 mM. Lastly, we confirmed the phenotypic influence of DAAO‐mediated H_2_O_2_ generation by counting the number of live cells after 24 hr of stimulation with D‐alanine. The Hela/mito‐DAAO cells were treated with D‐alanine concentration from 0 to 25 mM. Doses below 5 mM D‐alanine did not inhibit cell growth. Perturbation with D‐alanine to Hela cells devoid of DAAO system did not induce toxicity, consistent with the previous reports (Figure S8).^29^, ^40^ The findings shown in Figure 2c are consistent with previous results that a high dose of D‐alanine such as 25 mM killed cells through an apoptotic pathway.^29^

Next, we tested the functionality of the mitochondrial iNap sensor before implementation of experiments with DAAO system. First, we introduced an artificial oxidative stress by stimulating cells with 500 μM diamide, which was previously shown to minimally influence the fluorescence of iNap control sensor.^28^ We recorded an excitation spectrum with the emission wavelength centered at 515 nm, confirming a decrease of the ratio of 515 nm emission upon excitation with light centered at 415 and 488 nm as previously described (Figures 2d and S1E).^28^ Afterwards, we obtained the maximum fluorescence ratio of the iNap‐mito sensor. We stimulated Hela/iNap‐mito cells with 400 μM of NADPH with 0.05 mg/ml digitonin, and measured the change of fluorescence ratio every 20 s (Figure S1F). The effective fluorescence ratio was achieved by normalizing the fluorescence ratio of the iNap‐mito sensor to that of iNapC‐mito, which was designed to function as a control sensor that responds to pH (Figure 2e).^28^ Once the functionality of mito‐DAAO and iNap‐mito were validated, we constructed HeLa cell lines that stably expressed iNap sensors, either cytosolic or mitochondrial iNap variants, and transiently expressed mito‐DAAO to modulate mitochondrial H_2_O_2_ production rates.

### 2.2 Generation of mitochondrial H_2_O_2_ decreases mitochondrial, not cytosolic, NADPH pool

**FIGURE 3:**
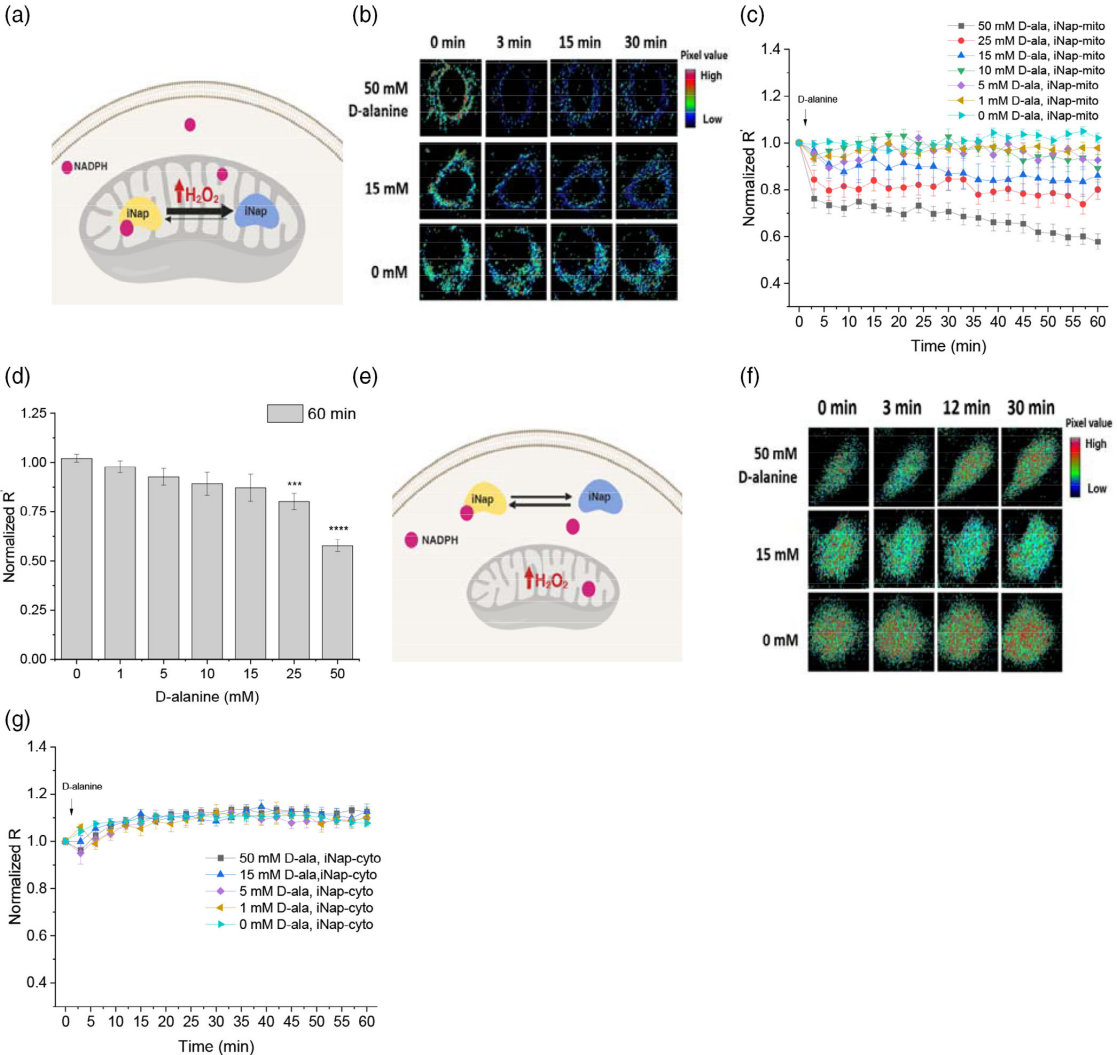
Generation of mitochondrial H_2_O_2_ via mito‐DAAO and measurement of compartmentalized NADPH via iNap‐mito or iNap‐cyto. (a) Schematics depicting a system that generates mitochondrial H_2_O_2_ via expression of mito‐DAAO and measures the mitochondrial NADPH pool using the iNap‐mito sensor. (b) Single cell images representing a fluorescence ratio of mitochondrial iNap sensors in response to D‐alanine treatment. Raw images were exported to MATLAB, processed to remove background signal, and the ratio of two images obtained from the 415 and 488 nm excitation channels was calculated. Individual pixel values were pseudo‐colored in range of 0 to 15, representing a dark‐blue (low) to red (high), whose values were used only for graphical visualization of cells. (c) Fluorescence readout (R^′^), normalized to that of initial value, was measured every 3 min after stimulation with D‐alanine ranging from 0 to 50 mM. Normalized R^′^ represent mean values of individual cells from at least two independent experiments ±*SEM*. (*n* = 21, 13, 12, 10, 14, 17, 21 cells from experiments with the 50 to 0 mM D‐alanine). (d) A relative change of normalized R^′^ from iNap‐mito at 60 min in response of D‐alanine addition. (e) Schematics representing a system with H_2_O_2_ generator in mitochondria and iNap‐cyto. (f) Time‐dependent change of a ratiometric fluorescence signal of cytosolic iNap sensor in response to D‐alanine treatment. (g) Normalized fluorescence ratio (R) was recorded with D‐alanine ranging from 0 to 50 mM. Values represent mean of individual cells from at least two independent experiments ±*SEM*. (*n* = 21, 11, 7, 8, 23 cells from experiments with 50 to 0 mM D‐alanine. A two‐tailed student's *t* test was used for statistical analysis with *p*‐values < .05 considered statistically significant (*p < .05, ***p < .001,  **** *p* < .0001)

To determine a threshold of D‐alanine concentration that would generate H_2_O_2_ in mitochondria and perturb the NADPH pool, we stimulated cells by adding 0, 1, 5, 10, 15, 25, 50 mM of D‐alanine and recorded the change of fluorescence readout (R^′^) of iNap‐mito (Figure 3a–c). The fluorescence ratios (R) were recorded every 3 min for 60 min. To account for the pH effect that could be introduced during the perturbation and generate an artificial fluorescence signal by the intrinsic property of circularly permuted yellow fluorescent protein, we performed parallel experiments with the pH sensor and normalized the fluorescence ratio of the iNap‐mito to that of iNapC‐mito (Figure S2A–C). Upon addition of D‐alanine below 25 mM, we observed the normalized fluorescence readout remained within 13% of that of the control sensor throughout the time span (Figure 3d). Above 25 mM, the fluorescence readout of the sensor decreased by 15% within 3 min and decreased by ~20% after 60 min. Upon addition of 50 mM of D‐alanine, the decrease of fluorescence readout was larger as it declined by 22% within 3 min and steadily decreased up to 40% in 60 min.

We previously demonstrated that an excessive generation of H_2_O_2_ via mito‐DAAO system could increase oxidation states of peroxiredoxin as well as glutathionylation of proteins under high concentration of D‐alanine, and suggested a threshold concentration of D‐alanine that triggered cellular toxicity to be between 15 and 25 mM for short perturbation times in Hela‐DAAO system.^29^, ^41^ Similarly, our data demonstrated that 25 mM D‐alanine was the threshold concentration that allowed a significant decrease of NADPH pools in mitochondria. Additionally, we assessed whether the absence of carbon source such as glucose or glutamine could influence the mitochondrial NADPH pool during high production of mitochondrial H_2_O_2_. In the absence of glucose, the normalized R^′^ from iNap‐mito decreased by nearly 50% compared with the samples that were treated with 25 mM D‐alanine with glucose (Figure S3A). In the presence of glutamine, the fluorescence readout was not statistically different, suggesting glucose metabolism as the primary source for maintenance of mitochondrial NADPH pool in 30 min.

Next, we explored whether the generation of mitochondria H_2_O_2_ influenced the cytosolic NADPH pool. We generated mitochondrial H_2_O_2_ via mito‐DAAO and recorded the fluorescence ratio of the cytosolic iNap sensor (Figure 3e–g). We expected that the fluorescence ratio of the cytosolic sensor would be maintained under low perturbation and change only at high perturbation. As predicted, addition of D‐alanine below 15 mM did not alter the fluorescence readout compared to that of the control. Interestingly, even at 50 mM of D‐alanine concentration, the fluorescence readout remained robust (Figures 3g, S2D, and S4A–D). This experimental data suggested that NADPH was impermeable to mitochondrial membrane as previously reported,^42^ and the cytosolic NADPH pool would not be depleted even under high production of mitochondrial H_2_O_2_. Additionally, we measured whether the DAAO‐mediated H_2_O_2_ production decreased the cellular NADPH pool using the luminescence‐based Promega NADP/NADPH‐Glo assay, and observed that the cellular NADPH/NADP^+^ decreased by up to 32% upon generation of mitochondrial H_2_O_2_ (Figure S3C–E).

### 2.3 Production of cytosolic H_2_O_2_ decreases cytosolic NADPH first followed by mitochondrial NADPH pool

**FIGURE 4:**
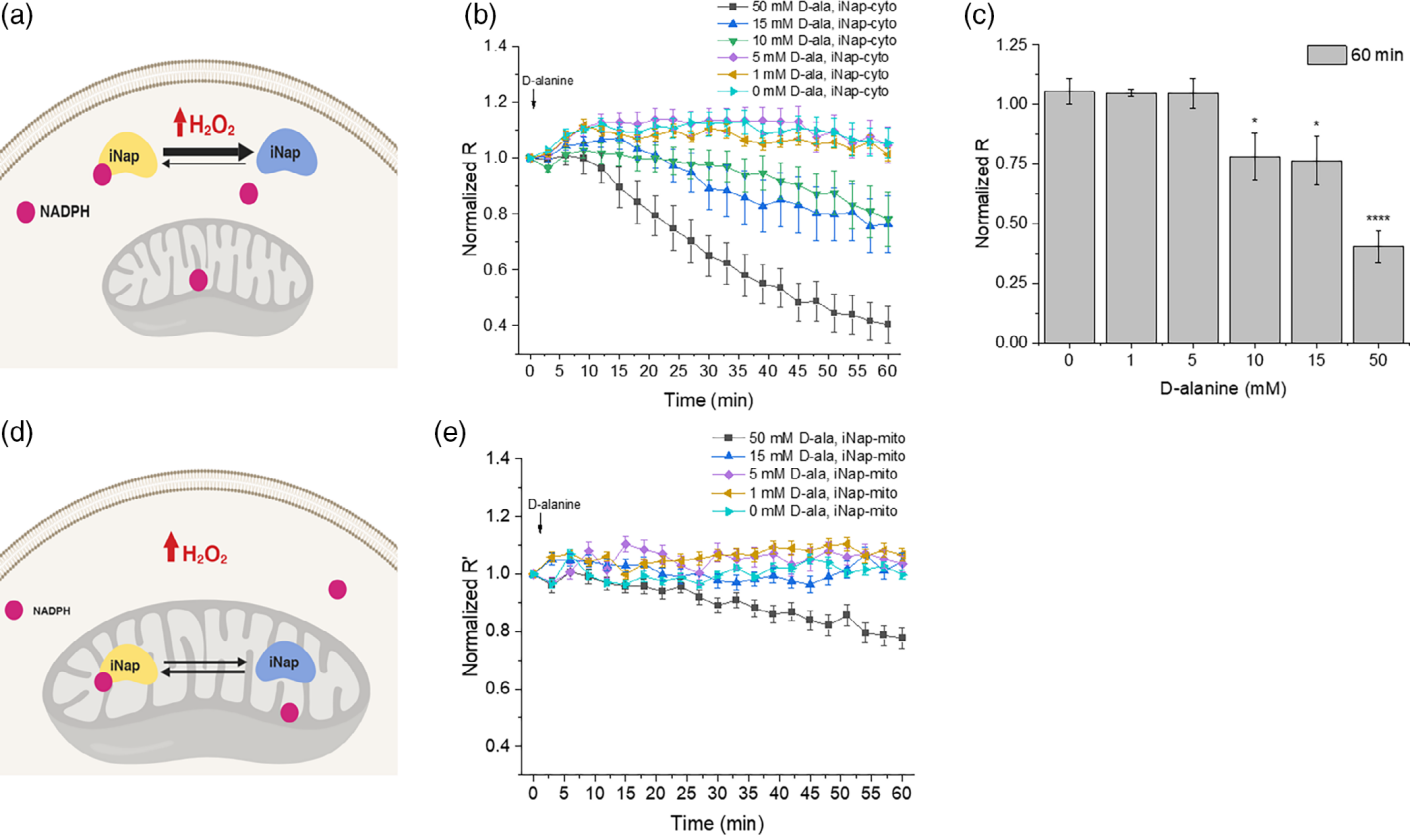
Production of H_2_O_2_ and measurement of NADPH in cytoplasm and mitochondria. (a) Schematic of cytosolic H_2_O_2_ generation by DAAO expressed in cytosol and NADPH measured by the cytosolic iNap sensor. (b) Normalized R from iNap‐cyto was monitored after stimulating cells with six different concentrations of D‐alanine. Values were recorded at every 3 min for an hour and represent mean of individual cells from at least two independent experiments ±*SEM*. (*n* = 10, 14, 14, 9, 9, 9 cells from experiments with 50 to 0 mM D‐alanine). (c) Normalized R from iNap‐cyto was measured at 60 min. (d) Schematic depicting the generation of cytosolic H_2_O_2_ by DAAO expressed in cytosol and the mitochondrial NADPH pools measured by iNap‐mito. (e) Normalized R′ from iNap‐mito was monitored after stimulating cells with a range of D‐alanine concentrations. Values were recorded every 3 min for an hour and represent mean of individual cells from at least two independent experiments ±*SEM*. (*n* = 39, 42, 12, 28, 17 cells from experiments with 50 to 0 mM D‐alanine). A two‐tailed student's *t* test was used for statistical analysis with *p*‐values < .05 considered statistically significant (*p < .05, *** *p* < .001, **** *p* < .0001)

As the fluorescence ratio of the cytosolic sensor was maintained under mitochondrial production of H_2_O_2_, we investigated whether the production of excessive H_2_O_2_ in the cytoplasm would in turn affect the mitochondrial NADPH pool. In this converse experiment, we generated cytosolic H_2_O_2_ via DAAO‐cyto by adding 0 to 50 mM of D‐alanine, and monitored the R or R^′^of iNap‐cyto and iNap‐mito in parallel (Figure 4a,d). When cells were challenged with D‐alanine below 5 mM concentration, the fluorescence ratio of the iNap‐cyto remained stable compared to that of control (Figures 4b, S2H). As the concentration increased above 10 mM of D‐alanine, the fluorescence ratio started to decrease over 60 min, with 50 mM D‐alanine lowering the fluorescence signal ~60% after 60 min (Figure 4b,c). The rate of decrease was higher with increasing dose above 10 mM. Unlike immediate decreases of mitochondrial NADPH pool as observed by iNap‐mito, iNap‐cyto revealed that the reduction of signal was delayed after 6 min under 50 mM D‐alanine perturbation. In parallel, we monitored the change of fluorescence readout of iNap‐mito under production of cytosolic H_2_O_2_ (Figure 4d,e). Interestingly, unlike the response of cytosolic iNap where the noramlized fluorescence ratio remained robust throughout different mitochondrial perturbations, the noramlized fluorescence readout of the iNap‐mito remained relatively constant throughout the cytosolic perturbation, except for a 50 mM D‐alanine perturbation (Figures 4e, S2E–G, and S4E–H).

### 2.4 Mitochondrial oxidative stress activates pentose phosphate pathway and glucose anaplerosis

**FIGURE 5:**
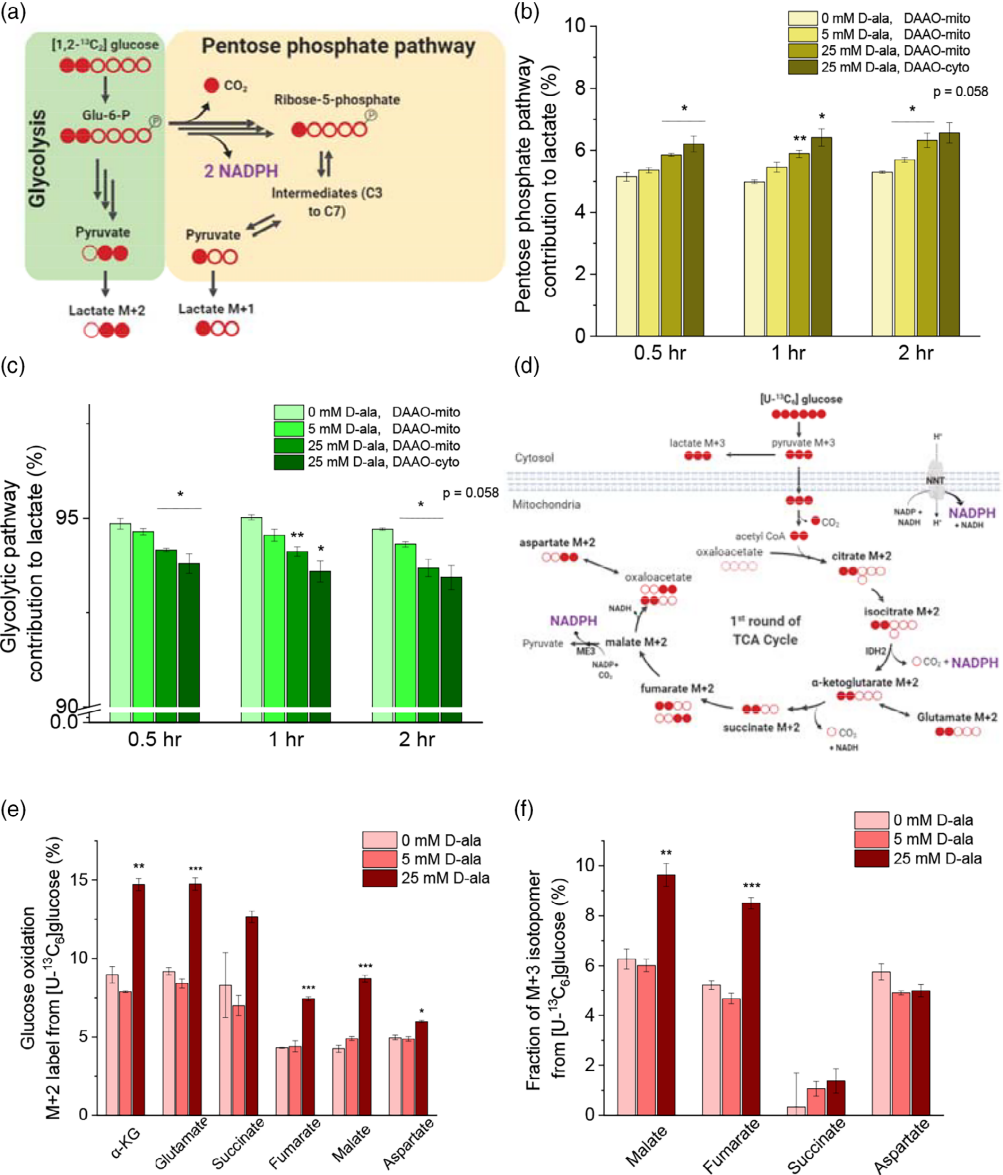
Mitochondrial oxidative stress increases fluxes through pentose phosphate pathway and glucose anaplerosis. (a) Schematic of the labeling pattern from [1, 2 − ^13^C_2_]glucose isotope tracer for measurement of a relative pathway strength between glycolysis and the pentose phosphate pathway. (b) Relative strength of pentose phosphate pathway is represented by the fraction of M + 1 of the sum of M + 1 and M + 2 lactate. (c) Glycolytic pathway activity is indicated by the fraction of M + 2 lactate. (d) Carbon transition map demonstrating oxidation of [U − ^13^C_6_]glucose tracer and the labeling pattern of TCA metabolites. (e) M + 2 and (f) M + 3 labeling patterns of the TCA metabolites are depicted under different concentrations of D‐alanine addition to Hela cells with mito‐DAAO at t = 2 hr

Whether the oxPP pathway can be triggered by the mitochondria‐derived oxidative stress for regulation of cytosolic or mitochondrial NADPH is largely unknown to our knowledge. To address this question, we used a [1,2‐^13^C_2_]glucose tracer and analyzed the relative pathway strength between glycolysis and the oxPP pathway.^43^, ^44^, ^45^ When the [1,2‐^13^C_2_]glucose tracer is converted to pyruvate through glycolysis, none or two carbons of pyruvate are labeled as^13^C. On the other hand, as the [1,2‐^13^C_2_]glucose is catabolized through the PP pathway, the first^13^C‐labeled carbon is lost at the 6‐phosphogluconate dehydrogenase reaction step along the 6GPD pathway, and thus only one is labeled, assuming the carbon shuffling and reductive pathway activity is minimal (Figure 5a).^43^ As the pyruvate pool rapidly equilibrates with that of lactate, we used lactate labeling data as a surrogate of that of pyruvate. Since the M + 0 lactate is a product from either glycolysis or the PP pathway, we calculated the fraction of M + 1 or M + 2 only. The fraction of M + 1 lactate represents the utilization of the PP pathway, whereas that of M + 2 lactate indicates the usage of glycolysis.

We hypothesized that flux though the PP pathway could increase when cells were challenged with excessive mitochondrial H_2_O_2_ generation rate. When a production rate of H_2_O_2_ in mitochondria increased beyond a certain threshold, H_2_O_2_ could diffuse out of mitochondria via aquaporin channels and the depolarization of mitochondria has been observed in longer time scales (t > 2 hr), leading to diffusion of H_2_O_2_ to the cytosol.^16^, ^29^ As a result, cytosolic NADPH may decrease, increasing the NADP^+^ level and activating glucose 6‐phosphate dehydrogenase.^46^ Alternatively, excessive generation of H_2_O_2_ in mitochondria could increase the NADP^+^ level in mitochondria and be transported to cytosol via indirect shuttles and activate the oxPP pathway.^47^, ^48^ Interestingly, even at mild perturbation such as 5 mM D‐alanine, the fraction of M + 1 lactate was increased by 4% compared with that of control within 30 min, 9% in 1 hr, and 7% in 2 hr (Figure 5b). For the treatment of 25 mM D‐alanine, the fraction was increased by approximately 14% in 30 min, 18% in 1 hr, and 19% in 2 hr. As a positive control, we supplemented 25 mM of D‐alanine to Hela cells that expressed the cytosolic DAAO and measured the fractions of M + 1 and M + 2 lactate. The cytosolic perturbation elevated the fraction of M + 1 lactate higher than that in mitochondria. The fraction of M + 1 lactate was increased by 20% in 30 min, 29% in 1 hr and 24% in 2 hr. As the fraction of M + 1 lactate increased in proportional to the D‐alanine concentration, the M + 2 lactate fraction decreased with increasing D‐alanine concentration. (Figure 5c). As a result, the increased ratio between the fractions of M + 1 and M + 2 lactate indicates a shift of some glycolytic flux to PP pathway during mitochondrial or cytosolic oxidative stress (Figure S9).

Next, we used a [U‐^13^C_6_]glucose tracer to determine whether the excessive production of mitochondrial H_2_O_2_ leads to a faster glucose oxidation rate and enhanced TCA cycle metabolism. As the [U‐^13^C_6_]glucose tracer is catabolized to pyruvate, the M + 3 pyruvate can be converted to either M + 2 acetyl‐CoA via pyruvate dehydrogenase complex (PDH) or M + 3 oxaloacetate via pyruvate carboxylase (PC). Through PDH reaction, the M + 2 acetyl‐CoA enters the TCA cycle and two carbons of the TCA cycle metabolites are subsequently labeled as^13^C (Figure 5d).^22^, ^43^ At high production rate of mitochondrial H_2_O_2_, we expected the glucose oxidation rate to increase so as to replenish TCA metabolites and support NADPH regeneration. Upon addition of 5 mM of D‐alanine, the fractional change of M + 2 metabolites was less than 10%. However, stimulation with 25 mM of D‐alanine increased the fraction of M + 2 metabolites by nearly 50% compared to that of non‐treated cells, suggesting a relative elevation of glucose oxidation via PDH reaction (Figure 5e).

Similar to the labeling profiles of M + 2 TCA metabolites, the addition of 25 mM of D‐alanine increased the fraction of M + 3 malate and fumarate (Figure 5f). The increase of M + 3 TCA metabolites can be attributed to second and higher rounds of TCA cycling or to utilization of PC reaction (Figure S5A–D). Heavily labeled mass isotopomers, such as M + 5 citrate, M + 5 α‐ketoglutarate and M + 4 succinate, represents the labeling pattern of the third and higher rounds of the TCA cycle. In a 2‐hr time scale, perturbed cells did not reach metabolic steady state and the fractions of M + 4 or M + 5 metabolites were shown less than 3% compared with the nontreated cells whose fraction reached to 10–15% after 24 hr (Figure S5E–J). As a result, the increased fraction of M + 2 and M + 3 metabolites suggested that mitochondrial reactions derived from the TCA cycle metabolites could be used as the main source of mitochondrial NADPH pools.

It is noteworthy that the DAAO system generates pyruvate as a byproduct and may interfere with central carbon metabolism. Upon the addition of 25 mM of D‐alanine, the total citrate pool increased and the fraction of M + 2 citrate was lower than that in the control cells. This could be due to the dilution effect of non‐labeled citrate introduced by the DAAO‐system and thus the fraction of M + 2 citrate could be underestimated as evidenced by the increase of the fraction of M + 0 citrate (Figure S5B). However, under low production of H_2_O_2_ with D‐alanine concentrations below 5 mM, the mass istopomer distribution pattern was similar across TCA cycle metabolites (Figure S5E–J). The non‐labeled pyruvate produced by DAAO could be catabolized to M + 0 acetyl‐CoA and participate in the TCA cycle by reacting with M + 2 aspartate from the end of a first round of TCA cycle. In this case, M + 1 isotopomers of α − KG, succinate, and fumarate would be expected to appear (Figure S6). If DAAO produced a substantial amount of nonlabeled pyruvate that subsequently fueled the TCA cycle, the fraction of M + 1 isotopomers would be expected to increase with higher perturbation, because M + 2 pyruvate derived from [U − ^13^C_6_]glucose could not theoretically generate M + 1 TCA cycle metabolites, assuming PDH as the major route of the anaplerotic pathways and correcting contributions of naturally occurring isotopomers. However, the fraction of M + 1 of all TCA cycle metabolites did not elevate throughout the perturbation, remained less than 3%, and were not statistically different from the control. Thus, these evidences suggest that DAAO‐mediated pyruvate would minimally interfere the central carbon metabolism under low perturbation.

### 2.5 Mathematical model estimates mitochondrial NADPH/NADP ^+^ to drop by 67‐fold under mitochondrial oxidative stress

With the dynamic experimental data obtained from the iNap sensors and the analysis of central carbon metabolism upon perturbation, we formulated a mathematical model to quantify the concentration of mitochondrial NADPH and NADPH/NADP upon varying production rates of H_2_O_2_. Based on the system of ordinary differential equations for redox species,^32^ we modified the model by updating kinetic vales of redox species for mitochondria and added new reaction terms such as (a) a permeability coefficient of D‐alanine, *P*, (b) a generation rate of hydrogen peroxide by DAAO, $k_{\mathrm{gen},H_{2}O_{2}}$, (c) a regeneration rate of NADPH, *k*_*gen*,*NADPH*_, and (d) a stress‐dependent NADPH flux coefficient, α (Figure 1, Table S1). Reasoning that NADPH could be transferred between cytosol and mitochondria via indirect metabolite shuttle systems above a certain threshold hydrogen peroxide generation rate in mitochondria,^47^, ^48^ we introduced the stress‐dependent NADPH flux, which was defined as a $\alpha \times v_{H_{2}O_{2}}^{\text{total}}$ . As the mitochondrial NADPH pool is decreased by high production rates of mitochondrial H_2_O_2_ flux, we expect an additional NADPH flux introduced in mitochondria to maintain the mitochondrial NADPH level.

**FIGURE 6:**
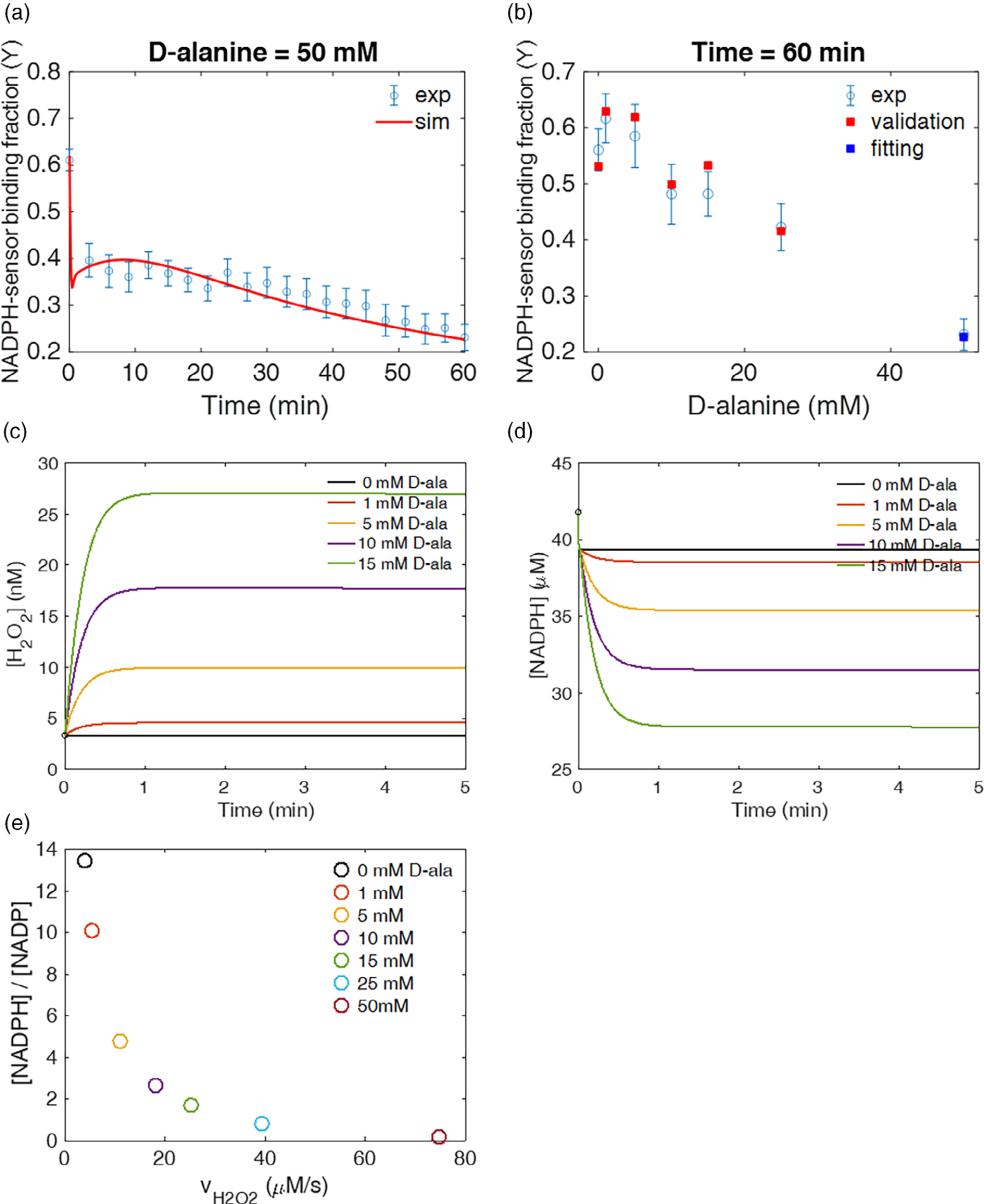
Computational model predicts mitochondrial NADPH/NADP^+^ ratio upon varying generation rates of H_2_O_2_ in mitochondria. (a) Unknown parameters were fitted to the experimental data of 50 mM D‐alanine to determine values consistent with the experimental data over 60 min timespan. (b) To assess the validity of these parameter values over a range of conditions, the fitted parameter values determined using the one condition in (a) were used to predict the binding fraction between NADPH and sensor (Y^model^) with different initial concentrations of D‐alanine and compared to the experimentally obtained value (Y^exp^) at t = 60 min. The blue square is the fitted condition, where the model and experiment must coincide. The red squares are not fit to the experimental data points. Upon different concentrations of D‐alanine used as input value, the model predicted (c) the intracellular H_2_O_2_ concentration in mitochondria, (d) NADPH, (e) and the NADPH/NADP^+^ ratio as a function of $v_{H_{2}O_{2}}^{\mathrm{tot}}$ at t = 60 min

We used a weighted least‐square minimization method based on time‐dependent experimental data ($Y^{\exp}=\frac{R^{\prime }-R_{\min}^{\prime }}{R_{\max}^{\prime }-R_{\min}^{\prime }}$) and model data ($Y^{\text{model}}=\frac{\text{NADPH}}{K_{d}+\text{NADPH}}$), which is derived from the binding kinetic equation between the sensor and NADPH. The initial concentration of redox species involved in this system were calculated as previously described (Table S2).^33^ The model data, including Y^model^, was obtained by solving the system of ordinary differential equations with 1,000 different sets of randomly‐chosen initial parameter values along with the basal NADPH concentration obtained from experiment and initial concentration of redox species found in literature (Table S2, Figure S7A‐B). For the experimental data, we used 21 data points obtained from the experiment with 50 mM D‐alanine stimulation as it represented the extreme condition per se mitochondrial oxidative stress (Figure 6a). Once optimized parameter values were determined, we assessed whether these values could simulate experimental data that were collected under six other conditions, each of which contained 21 time‐dependent data points (Figure 6b, S7C). Model‐predicted NADPH concentration and subsequently Y^model^ was compared to Y^exp^, demonstrating the robustness of the model as the values resided mostly within the mean± *SEM* range of experimental data at each time point.

The model predicted the overall generation rate constant of mitochondrial NADPH, k_gen,NADPH_, as 1.59 s^−1^ (1/s), and the steady‐state NADPH flux at the basal condition was estimated to be 4.05 μM/s. This value was within the same order of magnitude of the overall NADPH flux estimated from the FBA model in previous studies.^23^, ^49^ The stress‐dependent NADPH flux coefficient (α) was found to be 80.9. In the absence of α, the model prediction failed to predict the experimental data under higher perturbation (Figure S7D). The addition of D‐alanine increased the mitochondrial H_2_O_2_ level within the nM range with 15 mM D‐ala increasing the H_2_O_2_ level up to 7.5 times higher than the initial concentration of 3.3 nM (Figure 6c, S7E–H). Simultaneously, the NADPH level decreased inversely by the increase of H_2_O_2_ with 15 mM D‐alanine lowering NADPH pool ~34% (Figure 6d). When the model was simulated with 50 mM D‐alanine, the NADPH level dropped sharply within 0.5 min, increased again for ~27% within the next 8 min, and decreased again to a final concentration of 7.9 μM.

Sensitivity analysis further supported the increased role of stress‐dependent NADPH flux when the production rate of H_2_O_2_ was high (Table S3). The sensitivity of α to NADPH level was about three orders of magnitude lower than that of NADPH generation rate parameter below 15 mM D‐alanine perturbation, but it became within the same order of magnitude under 50 mM D‐alanine condition. Throughout the perturbation, the sensitivity of the NADPH generation rate parameter was the largest. At relatively lower perturbation (D‐alanine <15 mM), the sensitivity of H_2_O_2_ generation rate parameters ranked within top 5 along with the transport of D‐alanine. At higher perturbation (D‐alanine >25 mM), rate parameters such as TrxSS‐NADPH and H_2_O_2_‐Prx reactions become significant in controlling mitochondrial NADPH pools. Other fitted parameters included the permeability coefficient of D‐alanine, which was determined to be 6.92 × 10^−10^ cm/s and the generation rate coefficient of H_2_O_2_ by mito‐DAAO, which was evaluated to be 8.83 × 10^−2^ s^−1^ (Table S1).

Furthermore, the mitochondrial NADPH/NADP^+^ ratio decreased by 67‐fold when the generation of mitochondrial H_2_O_2_ increased by 19 times higher compared with basal rate (Figure 6e). The NADPH/ NADP^+^ ratio plays critical roles as multiple NADP dependent enzymes are reversible and a subtle change of ratio can alter the directionality of reactions, thereby switching the cellular metabolism.^12^, ^50^ Our model estimated the steady‐state mitochondrial NADPH/ NADP^+^ ratio at basal condition (0 mM D‐alanine) to be 13.4 (Table 1). This value is approximately 100‐fold lower than the whole NADPH/NADP ratio of live cells examined from classic literature, which reports that the ratio can reach as high as 1,000 under starved condition using a near equilibrium approximation.^26^ Under different initial concentrations of D‐alanine, the NADPH/ NADP^+^ ratio decreased inversely proportional to the total generation rate of H_2_O_2_ with 50 mM D‐alanine stimulation dropping the ratio down to 0.17, about two orders of magnitude lower than the basal ratio.

**TABLE 1:** Model prediction on the extent of NADPH/NADP^+^ under varying production rates of mitochondrial H_2_O_2_

D‐alanine (mM)	$v_{\mathrm{gen},H_{2}O_{2}}^{D-\mathrm{ala}}\left(\;\mathrm{\mu M}/s\right)$	$v_{\mathrm{gen},H_{2}O_{2}}^{\text{total}}\left(\;\mathrm{\mu M}/s\right)$	$\frac{\text{NADPH}}{\mathrm{NAD}P^{+}}$
0	0	4	13.4
1	1.4	5.4	10.1
5	7.1	11.1	4.8
10	14.1	18.1	2.6
15	21.2	25.2	1.7
25	35.3	39.3	0.8
50	70.7	74.7	0.2

*Note*: Instantaneous generation rate of H_2_O_2_ by DAAO‐mito system and the total generation rate was determined based on the simulation results with best‐fitted parameter values from experimental results. The ratio was calculated at t = 60 min.

## 3 DISCUSSION

NADPH is key to compartmentalized metabolic reactions and essential for defense against oxidative stress.^50^, ^51^ Here, we explored the distribution and dynamics of compartmental NADPH pools while systemically modulating generation rates of H_2_O_2_ in mitochondria. Previous work to assess mitochondrial NADPH metabolism was undertaken with deuterium‐labeled tracers and an expression of a reporter system of 2‐HG.^48^ These tracers have been useful in examining directionality of pathways that are present in both the cytoplasm and mitochondria, but a direct measurement of compartmentalized NADPH pools and evaluation of NADPH dynamics in living cells has been still lacking.

Imaging experiments revealed that the mitochondrial NADPH pool was sensitive to both mitochondrial and cytosolic oxidative stress whereas the cytosolic NADPH pool was minimally perturbed by the mitochondrial oxidative stress. Unlike the cytosolic NADPH pool that remained robust under mitochondrial oxidative stress, we observed the mitochondrial NADPH pool started to decrease under cytosolic oxidative stress. This could be due to diffusion of H_2_O_2_ via aquaporin or depolarization of mitochondrial membrane, causing a decrease of mitochondrial NADPH pools upon excessive production of cytosolic H_2_O_2_.^52^, ^53^, ^54^ Additionally, mitochondria lacks G6PD and 6PGD enzymes, which rapidly regenerate cytosolic NADPH via an allosteric regulation mechanism and are known to be major contributors of cytosolic NADPH pool. Thus, the regeneration of mitochondrial NADPH can be slower than the cytosolic production rate, resulting in a greater impact of compartment‐specific oxidative stress to the mitochondrial NADPH pool. Our work was based on Hela cells and this approach could be applicable to other cancer cell lines. We would expect similar trends across discrete cell lines, but the extent of NADPH buffering capacity and its influence on metabolic processes upon oxidative stress on different systems remains to be investigated.

^13^C glucose isotopic tracers demonstrated the activation of oxPP pathway and enrichment of TCA cycle metabolites during mitochondrial oxidative stress. As the oxPP pathway is considered a route for regeneration of cytosolic NADPH, we first expected the PP pathway would not be activated by a decrease of the NADPH pool in mitochondria. However, We observed the oxPP pathway activity was increased upon a relatively small decrease of mitochondrial NADPH, indicating that oxPP pathway may contribute to maintenance of NADPH pools under mitochondrial oxidative stress through The NADPH transport via metabolite shuttle systems such as malate/pyruvate, citrate/aKG or serine/glycine conversion.^47^, ^48^, ^55^, ^56^ Evaluation of shuttle systems involved in transport of NADPH between cytosol and mitochondria could provide insight on how NADPH can be redistributed between mitochondria and cytosol upon oxidative stress. Furthermore, upon mitochondrial oxidative stress, the mitochondrial NADPH pool was reported to be maintained by activation of serine metabolism as knockdown of serine hydroxymethyl transferase (SHMT2) decreased cellular NADPH/NADP^+^ and impaired tumor growth in MYC‐dependent cells.^57^ Investigation of the extent of serine metabolism in maintaining compartmentalized NADPH could provide additional axes of NADPH homeostasis from nutrients other than glucose and glutamine.

Additionally, it has been reported that an increase of mitochondrial NADP^+^ cause an increase of mitochondrial NAD^+^,^58^ indicating an interchange between NADP^+^ and NAD^+^ in mitochondria can facilitate transfers of the cofactors between the cytosol and mitochondria through shuttle systems. The activation of the oxPP pathway was immediate (< 30 min) under relatively low production rate of mitochondrial H_2_O_2_. On the other hand, we observed increased labeling patterns of TCA cycle metabolites under high production rates of mitochondrial H_2_O_2_, or so‐called mitochondrial oxidative stress. TCA cycle metabolites such as malate, iso‐citrate, or NADH cofactor can be used for regeneration of NADPH in mitochondria via ME3, IDH2, or NNT, respectively. NNT was suggested to play a major role in maintenance of mitochondrial NADPH as knockdown of NNT caused a disturbed NAD(P)H/NADP^+^ balance.^31^ Future imaging experiments with NADH/NAD^+^ sensors such as Peredox, SoNar, and Apollo in both mitochondria and cytoplasm could elicit insight on whether NNT enzyme plays a major role in the generation of mitochondrial NADPH.^59^, ^60^, ^61^ Alternatively, by utilizing serine tracers with mutant iso‐citrate enzymes, the activity of folate metabolism could be visualized under conditions of enhanced production of mitochondrial H_2_O_2_.^48^

The main objectives of our mathematical model was to evaluate the mitochondrial NADPH concentration and NADPH/NADP^+^ because this information was challenging to obtain solely from experimental data. As many NADPH dependent dehydrogenases in central carbon metabolism are reversible, the NADPH/NADP^+^ ratio could alter the directionality of reaction pathways depending on the ratio. Additionally, higher fluxes of reactive oxygen species such as H_2_O_2_ could transiently trigger signaling processes through redox relay via protein oxidation or induction of transcriptional factors such as Nrf2, where NADPH is involved in this regulatory network via electron donors to support the antioxidant pathways.^62^, ^63^, ^64^

Several genome‐scale flux balance analysis (FBA) models have been recently adopted to predict NADPH flux at steady‐state and assessed metabolic reactions that contributed NADPH pools with constraints obtained from experimental results such as metabolite intake and uptake rates or proteome bioinformatics data.^23^, ^37^ Although these models demonstrated feasibility of identifying metabolic reactions that contributed most to NADPH pool such as folate cycle pathway, PP pathway or IDH reaction, the model was limited by the steady‐state assumption and difficulty of quantifying concentration of species involved in the model. The NADPH/NADP^+^ ratio and free pool sizes are known to vary depending on subcellular organelles, nutritional or stress conditions.^26^, ^50^ The FBA or metabolic flux analysis (MFA) could not estimate the change of NADPH pool size or its ratio as a function of time.

The kinetic model employed in our study provides time‐dependent changes of NADPH concentration and subsequently mitochondrial NADPH/NADP^+^, which cannot be easily determined by experiments or FBA or MFA models. Based on the model simulation, we estimated the mitochondrial NADPH/NADP^+^ could decrease by nearly 10‐fold when mitochondrial H_2_O_2_ flux increased 6.3 times greater than the basal rate. This condition could be an artificial threshold line as we observed a statistically significant decrease of mitochondrial NADPH pool from the imagining experiments. Classic literature calculated the NADPH/NADP^+^ under different diet conditions of rat liver assuming near‐equilibrium states of cytosolic malic or isocitrate dehydrogenase enzymes, and indicated that the cytosolic ratio could increase up to 8.8 times higher under starved condition.^26^ When the generation rate of mitochondrial H_2_O_2_ increased by nearly 15 times higher, the model predicted the ratio could drop to 67‐fold. Additionally, the increased sensitivity of the stress‐dependent NADPH flux coefficient (α) under stronger perturbations suggested an importance of additional NADPH production required to maintain mitochondrial NADPH pool. This finding suggested an increased role of NADPH transport from cytosol to mitochondria via metabolite shuttle systems. Whether cells would prefer specific shuttle systems remain to be determined.

Further, considering the model‐estimated production rates of mitochondrial H_2_O_2_ and the stoichiometry of DAAO reaction (O_2_ + D − alanine → H_2_O_2_ + pyruvate), the generation of pyruvate by the DAAO system is expected to be less substantial than intracellular pyruvate pools at lower perturbation. TCA metabolites are estimated to be equilibrated at milli‐ to micro‐molar ranges via dehydrogenase enzymes,^26^ whereas the generation rate of mitochondrial H_2_O_2_ by DAAO‐system is expected to be in the nM/s range at low perturbation. The intracellular concentration of mitochondrial H_2_O_2_ was expected to be in low nano‐ to high pico‐molar ranges,^65^ which would be orders of magnitude lower than TCA cycle metabolites. Future work on computational model involves updating kinetic parameters by measuring other redox species such as glutathione and thioredoxin pairs in mitochondria, and integrating cytosolic and mitochondrial models using transport reaction terms of redox species such as NADPH and H_2_O_2_.

## 4 CONCLUSIONS

**FIGURE 7:**
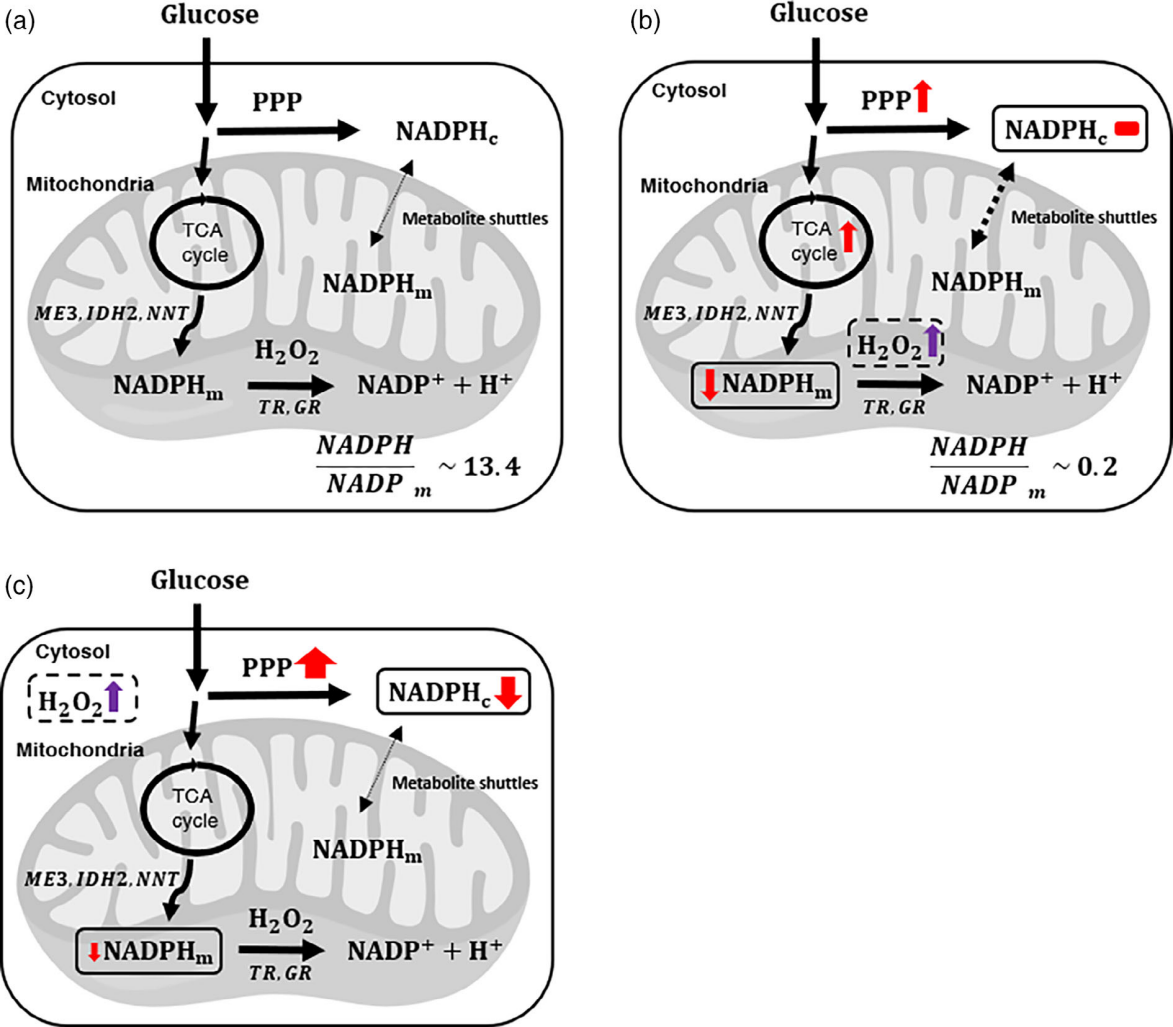
Proposed models for glucose metabolism regulating compartmentalized NADPH pools upon mitochondrial or cytosolic oxidative stress. Overview of major reaction pathways for generation and consumption of mitochondrial and cytosolic NADPH, and cellular responses at (a) the baseline, or at conditions when cells were challenged with excessive production of (b) mitochondrial H_2_O_2_, or (c) cytosolic H_2_O_2_

Combining iNap sensors, ^13^C glucose isotopic tracers, and a mathematical model, we provide insight on mitochondrial NADPH metabolism under varying production rates of mitochondrial H_2_O_2_. Mitochondrial oxidative stress lowers the mitochondrial NADPH pool, activating the pentose phosphate pathway (PPP) and glucose anaplerosis to maintain NADPH pools (Figure 7). Cytosolic NADPH pool is robust during mitochondrial oxidative stress, whereas mitochondrial NADPH pool is influenced by cytosolic oxidative stress. These observations indicate compensatory changes of glucose metabolism to maintain NADPH homeostasis in response to compartmentalized oxidative stress.

Additionally, the mitochondrial NADPH/NADP^+^ ratio is estimated to change for nearly two orders of magnitude under mitochondrial oxidative stress. Incorporation of our data to metabolic flux analysis (MFA) or FBA as additional constraints will provide insight on how the ratio can influence the reaction directions of metabolic pathways such as reductive glutamine metabolism or serine catabolism. To which extent mitochondrial or cytosolic NADPH/NADP^+^ impact reaction directions remains yet to be determined. Finally, as modulating oxidative stress has been suggested as an anticancer strategy,^66^ our results motivate further investigation of compartmentalized and particularly mitochondrial NADPH production pathways that can be used as targets that differentially affect transformed and normal cells or cancer cells with disparate lineages.

## 5 METHODS

### 5.1 Plasmid reconstruction of mito‐iNap, mito‐iNapC, iNap, and iNapC

Mito‐iNap and mito‐iNapC fragments were prepared via an IDT gBlocks Gene Fragments service with NheI and EcoRI attached on 5′ and 3′ ends after following the sequence information provided by the Tao et al. The sensor fragments were subcloned into pLJM1‐EGFP lentiviral transfer plasmid using NheI and EcoRI restriction enzyme sites. To construct the iNap sensor without the mitochondria localization sequence, pLJM1‐mito‐iNap plasmids were amplified to pLJM1‐iNap using primers that amplify only the iNap region. Subsequently, a site‐direct mutagenesis were implemented using a Q5 site‐directed mutagenesis kit (NEB, #E0554S). The iNapC were constructed by removing mitochondrial localization sequence from the mito‐iNapC construct. Final sequences of the sensor constructs were confirmed by the DNA sequencing service of Quintara bioscience.

### 5.2 Creation of cell lines stably expressing mito‐iNap, mito‐iNapC, iNap33, and iNapC

HEK293 FT cells were seeded at 7.5 ×10^5^ cells per 35 mm well in 6 well plates (Corning, VWR 29442–042) for 2 days until 70–90% confluency. pLJM‐1 vectors encoding appropriate sensors were co‐transfected with the packaging plasmids pMD2.G and Pax2 vectors at a 3:1: 2 ratio for a total of 5 μg plasmids and 10 μg of Lipofectamine 2000 in OptiMEM medium overnight. Next morning, the media was replaced with 1 ml of Dulbecco's modified Eagle's medium (DMEM; Lonza) supplemented with 10% fetal bovine serum (FBS; ATCC) and the media was collected every 24 hr for 2 days. The collected media was centrifuges at 500 g for 5 min and the supernatant was collected and stored at −80C freezer. After virus containing supernatant was prepared, Hela cells were seeded at 3.5 × 10^5^ cells per 35 mm well in 6 well plates for 2 days until 70–90% conflunecy. Then, 1 ml of virus‐containing supernatant was added to wells containing Hela cells with 6 μg/ml of polybrene. After 3 days of infection, cells from each well were expanded to 10 cm dish with 6 μg/ml puromycin selection media for about 7 days until 70–90% confluency.

### 5.3 Immunofluorescent analysis of Hela cells transiently transfected with mito‐DAAO‐FLAG

Hela cells, expressing mito‐DAAO‐FLAG in 6‐well plates at a cell density of ~35,000 cells per well, were fixed with 4% paraformaldehyde and permeabilized with 0.1% Triton X‐100 in PBS for 30 min at room temperature and blocked with an Odyssey Blocking buffer (Li‐Cor) in PBS with a 1:1 ratio for 30 min. Cells were stained with AlexaFluor 488 conjugated FLAG‐tag, monoclonal antibody (Product # MA1‐142‐A488) at a dilution of 1:100 for an hour at room temperature. The sequence of Flag‐tag is DYKDDDDK and it can be appended to any recombinant gene so that the protein of interest it encodes can be easily labeled within the cell to check for expression and localization. Nuclei were stained with the DAPI. Images were captured on an Olympus IX‐81 microscope with a CCD camera at 20X magnification.

### 5.4 Transient transfection of D‐amino oxidase oxidase (DAAO) and DAAO‐FLAG

Hela cells stably expressing appropriate iNap sensors were seeded at 1.75 ×10^5^cells per 35 mm well in 6 well plates or 35 mm single well dish. After 2 days, the media was replaced to 1 ml of OptiMEM medium and transiently transfected with the pLJM1‐mito‐DAAO plasmid or pLJM1‐FLAG‐mito‐DAAO using the Lipofectamine 2000 in OptiMEM medium with a total DNA concentration of 2 μg.

### 5.5 Generation of mitochondrial hydrogen peroxide using the DAAO

After 18 hr of transient transfection, the media were replaced to RPMI 1640 without phenol red media supplied with flavin adenine dinucleotide at 5 μM and equilibrated for at least 45 min in the 37°C incubator with 5% CO_2_. The dish was set up on the microscope stage and the appropriate concentration of D‐alanine was added.

### 5.6 Cellular imaging using fluorescence microscopy

The fluorescence emission signal was recorded using an inverted IX81 widefield fluorescence microscope (Olympus) with a ×20 objective lens and Prior Lumen 2000 lamp. The Chroma 415/30 nm and a Semrock 488/6 nm excitation filters were used and a Semrock 525/40 nm filter was used for the emission fitler. The exposure time was 300 ms and 10% lamp intensity was used. A in‐built multidimensional setup was used for automated time‐course measurement. Images were captured every 20 s, 1 or 3 min and exported to either ImageJ or MATLAB 2016a for post image processing.

### 5.7 Image analysis of iNap sensors

Backgrounds of short (415 nm) and long (488 nm) wavelength images were subtracted using a rolling ball algorithm from ImageJ. Long‐wavelength images were converted to 32 bit and a threshold of one was applied to minimize artifact. The pixels values of the 415 nm filters were divided those of the 488 nm. Individual cells, neither too bright not too dim, were randomly selected and the mean fluorescence intensity of region of interest was calculated. All the fluorescence emission ratios were recorded as the mean ± *SEM*. The images were created using the image processing algorithm in MATLAB 2016a with pseudo colors.

### 5.8 Cell culturing and isotope labeling experiments

For isotopic tracer labeling experiments, 1.75 × 10^5^ cells were seeded onto 6 well plates with 2 ml of DMEM (Lonzo, 12001–568) containing 10% dialyzed FBS (ATCC 30–2020) and 6 μg/ml puromycin (Sigma), and cultured for 2 days. The cells were transiently transfected with 2 μg of DAAO‐mito and 4 μl of lipofectamine 2000 following the manufacturer's protocol. For isotopic labeling experiments, glucose‐free RPMI (Thermofisher, 11879020) was supplemented to a total 11.1 mM of 100% composition [1,2‐^13^C_2_]glucose (Cambridge Isotope Labs) or [U‐^13^C_6_]glucose (Cambridge Isotope Labs). After replacement of the culture media to the RPMI media with isotopic tracers, the plates were placed in humidified CO_2_ incubator at 37°C for 2 hr before performing experiments.

### 5.9 Metabolite extractions and GC/MS analysis

Extraction and analysis methods were followed as previously described.^67^ At 70–80% confluency, media was aspirated and intracellular metabolites were quenched with −20°C methanol. Internal standards were added and cells were scraped from the well and resuspended in the centrifuge tube. −20°C chloroform was added, centrifuged at a max speed in 4°C. The polar phase and nonpolar phase solvents were obtained in separate tube, and the polar phase solvents were dried until completely evaporated. The polar metabolites were derivatized by addition of Methoxyamine in pyridine (MOX) at 40°C for 1.5 hr and *tert‐*butyldimethylchlorosilane (TBDMS) was subsequently added and dried at 60°C for 1 hr. The derivatized samples were injected on the GC/MS. GC/MS analysis was complete using an Agilent 6890 GC connected with a 30‐m DB‐35MS capillary column with an Agilent 5975B MS operation under electron impact ionization at 70 eV. Then, 1 μl of the sample was injected at 270°C using helium as the carrier gas at a flow rate of 1 ml per min. The GC oven temperature was fixed at 100°C for 3 min and increased to 300°C at 3.5 per min. The detector was set in scanning mode and the mass‐to‐charge ratio was measured in range of 100–1000 m/z.

### 5.10 Computational methods

#### 5.10.1 Overview of kinetic model

The purpose of this model is to quantify the mitochondrial NADPH and NADPH/NADP^+^ under varying production rates of H_2_O_2_ using a system of ordinary differential equations (ODEs). The source of input data includes the fraction of sensor readout described in the later section and the well‐established literature values for kinetic constants and initial concentrations. First, we created a system of ordinary differential equations for redox species, the kinetic model first reported by Adimora et al.^32^ With the model as a framework, we updated values of rate constants and initial concentration of redox species found in mitochondria. In regards to the regeneration rate for NADPH in mitochondria, we set a first order kinetic equation that represents major enzymatic reactions for NADPH production such as IDH2, ME3, NAD^+^transhydrogenases (NNT), and methylene tetrahydrofolate dehydeogenase 2 (MTHFD2).^68^, ^69^

We included the transport and degradation rates of glutathione, removing the glutathione synthesis rate as it was exclusively formed in the cytoplasm.^70^ The thioredoxin influx and degradation rates were removed as their rates were indicated to be three orders magnitude lower than other redox reactions, thereby its sensitivity to the system low.^32^ The generation rate of mitochondrial H_2_O_2_ at the basal level was determined from references, which reported values that were within the same order of magnitude.^71^, ^72^, ^73^ The generation rate of was D‐alanine we introduced would only be catabolized by the DAAO we expressed in mitochondria and not by other enzymatic reactions based on the fact that HeLa and most other human cells lack naturally occurring DAAO.^40^, ^74^ Thus, we set cytosolic D‐alanine to be constant. Because of this assumption and for simplification of mathematical model, we can set only one D‐alanine transport rate that separates D‐alanine in media and D‐alanine in mitochondria. Lastly, we included a stress‐dependent NADPH flux that was defined as a function of the total mitochondrial H_2_O_2_ flux.

The initial concentration of oxidized redox species in mitochondria were calculated based on the steady state approximation with the molar balance equations as described before.^33^ Unless noted, we assumed rate constants of redox reactions in mitochondria are within the same order of magnitude of those in cytosol and thus used accordingly as listed in Table 1. For the initial concentration of mitochondrial NADPH concentration, we converted the fluorescence ratio of the sensor to NADPH concentration based on the digitonin based calibration experiments (Figure S1F) as described previously.^28^ The average concentration of NADPH in mitochondria was determined to be 41.8 μM based on the fluorescence images of 243 single cells (Figure S7A).

#### 5.10.2 Quantification of NADPH

We quantified the mitochondrial NADPH level by calculating the fraction of sensor readout (Y^exp^) and equating it to the fraction of sensor‐NADPH complex (Y^*model*^), which are expressed as follows:

$$Y^{\exp}=\frac{R^{\prime }-R_{\min}^{\prime }}{R_{\max}^{\prime }-R_{\min}^{\prime }},Y^{\text{model}}=\frac{\text{NADPH}}{K_{d}+\text{NADPH}}$$

*R*^′^ is an effective fluorescence signal obtained by $\frac{{\left.R_{415\mathrm{nm}/488\mathrm{nm}}\right|}_{\text{iNap}-\text{mito}}\;}{{\left.R_{415\mathrm{nm}/488\mathrm{nm}}\right|}_{\text{iNapC}-\text{mito}}}$.^28^ $R_{\max}^{\prime }$ was obtained by permeabilizing Hela cells expressing mito‐iNaps with an optimized concentration of 0.05 mg/ml of digitonin and incubating with 400 μM NADPH (Figure S1F). To minimize artifact effects such as leakage of sensor, we have used the control NADPH sensor with no binding affinity to NADPH in parallel and normalized the sensor readout to that of control sensor.^28^ To control the permeabilization of mitochondrial NADPH, we varied the concentration of digitonin from 0 to 1 mg/ml in presence or absence of NADPH in context of our experiments (Figure S1F). As NADPH sensor was localized to mitochondria, NADPH sensor fluorescence signal did not change unless we introduced a threshold digitonin concentration. We observed the rise of signal was dependent on time‐scale as lower concentration (0.05 mg/ml) allowed increase of signal at later time‐points while higher concentration (0.1 mg/ml) allowed permeabilization effect in earlier time‐point such that we observed a sharp increase of signal followed by steep decrease of signal potentially due to the leakage of sensors.

$R_{\min}^{\prime }$ was determined by addition of 100 mM D‐alanine to Hela cells expressing DAAO and mito‐iNaps. Due to the presence of antioxidant network present in cells, the $R_{\min}^{\prime }$ could be underestimated. Thus, we compared the quantified NADPH concentration to that of reference ^28^, where the estimation of intracellular NADPH level from different variants of iNap sensors was consistent with in vtiro evaluation, and our estimates of free NADPH value fell within 5% of that determined in the reference (S7B). For Y^*model*^, NADPH represents concentration of mitochondrial NADPH and K_d_ is the dissociation constant of the iNap3 sensor, which is 25.2 μM taken from the literature.^28^ Y^*model*^ is derived based on the equation of binding interaction between the ligand and the sensor with one to one stoichiometry.

#### 5.10.3 Objective function for parameter evaluation

With time‐course measurement of NADPH level in terms of concentration, we fitted model parameters by minimizing an objective function that calculates the sum of squared difference between predicted and observed values as follows:

$$Z\left(\theta \right)=\sum_{i=1}^{N_{\exp}}\sum_{k=1}^{N_{k}}w_{i}\left(t_{k}\right){\left[y_{i}^{\text{pred}}\left(t_{k};\theta \right)-y_{i}^{\mathrm{obs}}\left(t_{k}\right)\right]}^{2}\;$$

where *y*^*obs*^ is the experimentally observed NADPH concentration, *w*_*i*_(*t*_*k*_) is the 1/σ_*i*_(t_*k*_)^2^, σ is the *SEM*, *N*_*k*_ is the number of data points taken for duration of 60 min with time interval of 3 min, and *N*_*exp*_ is the number of experiments with five different conditions.

#### 5.10.4 Sensitivity analysis

Using the finite approximation methods, we implemented a sensitivity analysis to the NADPH level by every parameters in the reaction model.^75^ The equation of sensitivity analysis is as follows:

$$s_{i}=\frac{\partial C_{\text{NADPH}}}{\partial \theta_{i}}=\frac{C_{\text{NADPH}}\left(\theta_{i}+∆\theta_{i},t\right)-C_{\text{NADPH}}\left(\theta_{i},t\right)}{∆\theta_{i}}$$

where s_*i*_ represents the sensitivity to the *θ*_*i*_ model parameters and *C*_*NADPH*_ is the concentration of NADPH. Parameters were varied by 10% and the time was evaluated at 3 min. As the parameter values vary by orders of magnitude, we normalized the sensitivity to *C*_*NADPH*_(*t*) and *θ*_*i*_. The final sensitivity equation is as follows:

$$\bar{s_{i}}=\frac{\partial C_{\text{NADPH}}/C_{\text{NADPH}}\;}{\partial \theta_{i}/\theta_{i}}$$

All the normalized sensitivities were evaluated at corresponding D‐alanine perturbations and the top 5 most sensitizing parameters were reported (Table S5).

### 5.11 Quantification and statistical analysis

All results are represented as mean ± *SEM* of at least three biological replicates, unless indicated.

A two‐tailed student's *t* test was used for statistical analysis with *p*‐values < .05 considered statistically significant (*for .01 < *p* < .05,  ** .001 < *p* < .01,  *** .001 < *p* < .001, and **** *p* < .0001).

## DECLARATION OF INTERESTS

The authors claim no competing interests.

## AUTHOR CONTRIBUTIONS

Hadley D. Sikes, Gregory N. Stephanopoulos, and Sun Jin Moon conceived the study; Sun Jin Moon designed and performed most of the experiments and work with the kinetic model; Wentao Dong aided isotopic tracer experiments and interpretation of results; Sun Jin Moon wrote the paper with input from Hadley D. Sikes, Gregory N. Stephanopoulos

6

## Supporting information


**Appendix** S1: Supporting Information

## Data Availability

The MATLAB code for running the kinetic model for clearance of mitochondrial H_2_O_2_ are freely available at the following GitHub URL: https://github.com/sunjjmoon/runMitoH2O2clearanceModel
