# Supplementary material for: Oxidative pentose phosphate pathway and glucose anaplerosis support maintenance of mitochondrial NADPH pool under mitochondrial oxidative stress
Source: Bioeng Transl Med. 2020 Sep 8;5(3):e10184. doi: 10.1002/btm2.10184 (PMC7510474; doi:10.1002/btm2.10184)
Supplement: Supplementary file 1 — Appendix S1: Supporting Information [file BTM2-5-e10184-s001.zip › BTM2_10184_Moon SI-v2 clean.docx]

**Supplemental Information**

**
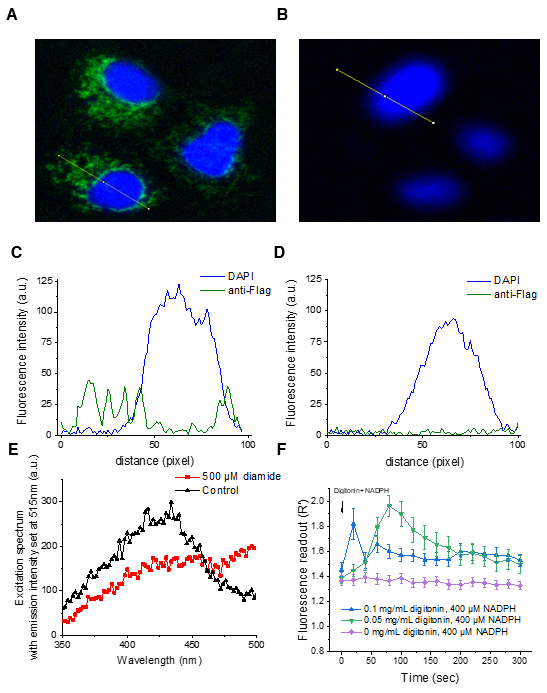
**

**Figure S1, related to Fig 2.** Localization of the DAAO-mito construct was confirmed by comparing the fluorescence intensity of FLAG and DAPI channel in individual pixel. Fluorescence intensity was evaluated to HeLa cells that were transiently transfected with FLAG-DAAO-mito **(A, C)** or none **(B, D)**. The blue represents fluorescence intensity from the DAPI-staining channel and the green from the FLAG-tag channel. 50 pixels are equivalent to 16.125 µm based on MetaMorph Microscopy analysis software. **(E)** The excitation spectrum with the emission set at 515nm was evaluated using a plate reader. $3.5\times{10}^{5}$ cells were plated in the 6-well plate a day before the experiment. The excitation spectrum was developed before and after the addition of 500 uM diamide. The maximum fluorescence was obtained at 416 nm and the minimum fluorescence was observed at 490 nm. **(F)** A time-course measurement of fluorescence readout ($R^{'}$) at different concentration of digitonin. A 0.1 mg/mL concentration permeates the membrane fast such that the response was immediate. A 0.05 mg/mL digitonin still permeated the mitochondrial membrane but a slower time scale. 350,000 cells were seeded to each well of 6-well plates a day before the experiment. Data $\pm$ S.E.M with n= 27, 26, 22 cells, respectively.


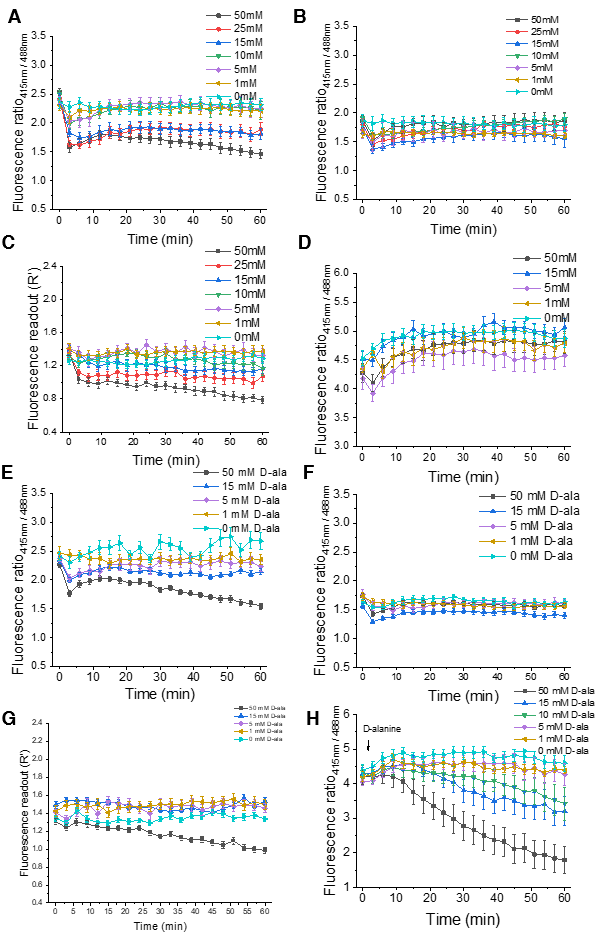


**Figure S2, related to Fig 3 and 4.** Fluorescence ratios of iNap sensors upon generation of hydrogen peroxide via DAAO-mito by stimulating cells with varying concentration of D-alanine. **(A)** Fluorescence ratio of iNap-mito, **(B)** iNapC-mito, **(C)** ratio of iNap-mito to iNapC-mito, which is fluorescence readout $R^{'}$, and **(D)** iNap-cyto. **(E)** Fluorescence ratios of iNap sensors upon generation of $H_{2}O_{2}$ via DAAO: Fluorescence ratio of iNap-mito, **(F)** iNapC-mito, **(G)** ratio of iNap-mito to iNapC-mito, which is fluorescence readout $R^{'}$, and **(H)** iNap-cyto.


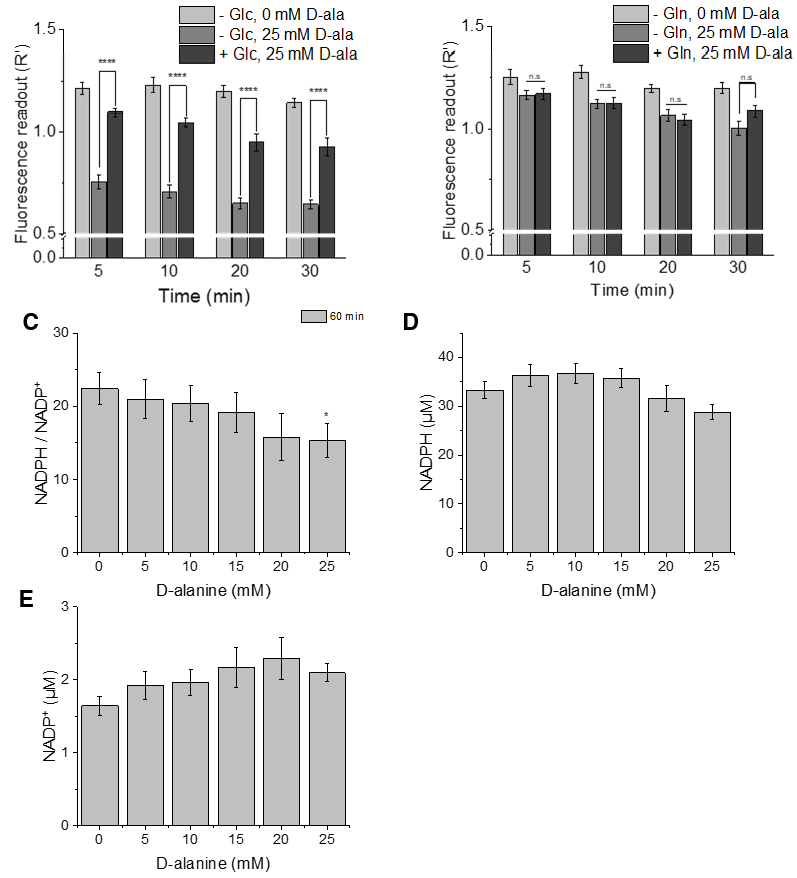


**Figure S3, related to Fig 3 and 4.**  Normalized $R^{'}$ from iNap-mito was recorded upon generation of mitochondrial hydrogen peroxide in media with absence or presence of glucose or glutamine. $3.5\times{10}^{5}$Cells were plated into 6 well plates for two days before the transient tracnsfection with DAAO-mito. The fluorescence ratio was recorded every minute in media with or without **(A)** glucose, and **(B)** glutamine. (**C)** The whole cellular NADPH/NADP ratio, **(D)** NADPH, and **(E)** NADP^+^ were measured using the luminescence-based enzymatic assay by Promega. Cells were cultured in 6-well plates as described in methods with intracellular NADPH and NADP^+^ concentration estimated assuming volume of Hela cells were 4.188 pL and the number of cells in each well of 96-well plates was 21,000 ^38^. Values were determined following manufacturer’s protocol. Mean and error bars represent five biological replicates with three technical replicates each. A two-tailed student’s *t-*test was used for statistical analysis with p-values $<$ 0.05 considered statistically significant ($*P<0.05, ***P<0.001, ****P<0.0001$).


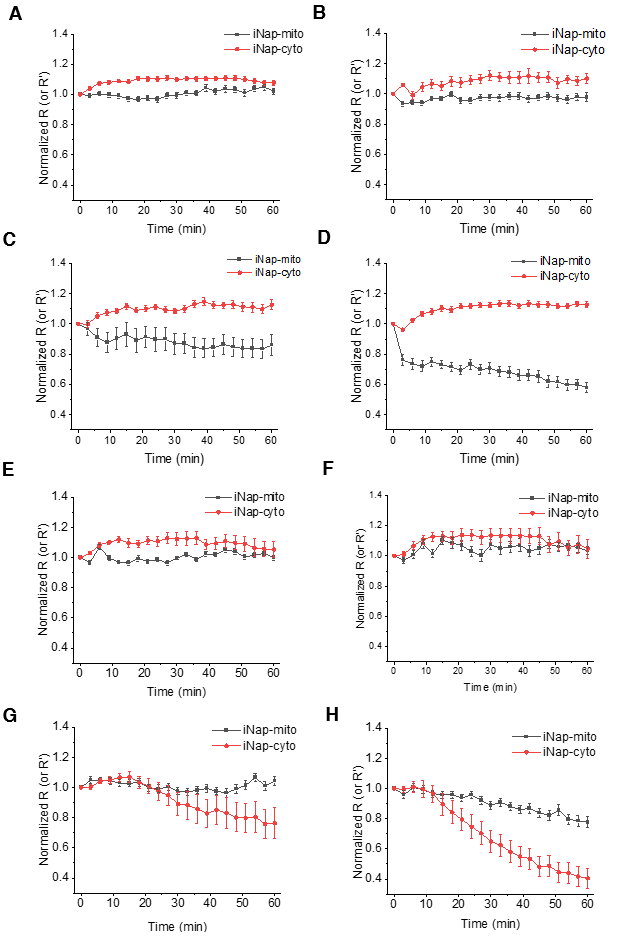


**Figure S4, related to Fig 3 and 4.** Comparisons between the normalized R for iNap-cyto and normalized $R^{'}$ for iNap-mito upon generation of $H_{2}O_{2}$ via mito-DAAO. Concentration of D-alanine varied from **(A)** 0, **(B)** 1, **(C)** 15, to **(D)** 50 mM. Similarly, normalized R for iNap-cyto and normalized $R^{'}$ for iNap-mito were compared upon generation of $H_{2}O_{2}$ via DAAO. Concentration of D-alanine varied from **(E)** 0, **(F)** 1, **(G)** 15, to **(H)** 50 mM. The black line represents iNap-mito and the red line indicates iNap-cyto.


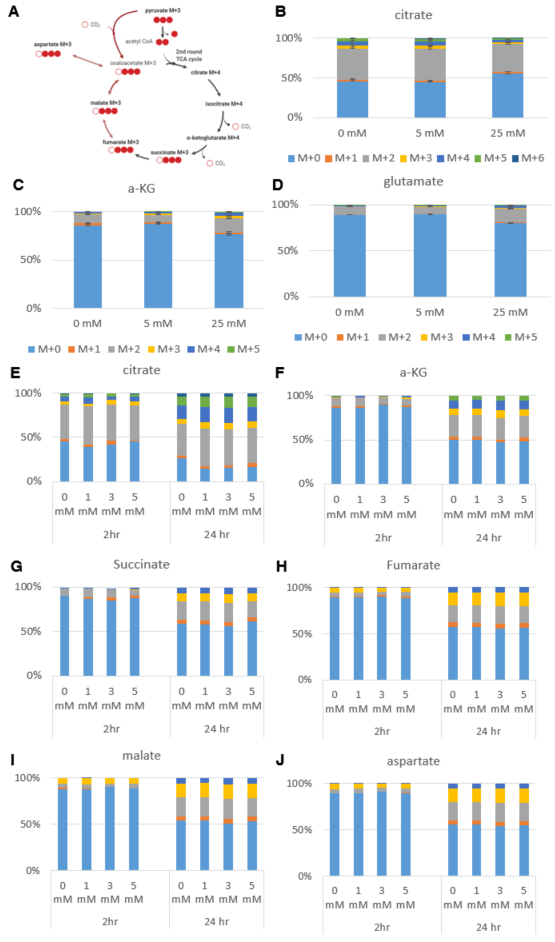


**Figure S5, related to Fig 5. (A)** Schematics representing the utilization of pyruvate to oxaloacetate via pyruvate carboxylase from [U-${{}^{13}C}_{6}]\mathrm{glucose}$. When the 2^nd^ TCA cycle undergoes from the first cycle of TCA, the M+4 metabolites would be observed. Citrate could be made as M+5 if the M+3 oxaloacetate is combined with M+2 acetyl-CoA. **(B)** The mass isotopomer distribution (MID) of citrate is depicted. The M+0 fraction is increase up to 10 % under 25 mM D-alanine perturbation, suggesting an effect of dilution introduced by non-labeled pyruvates from the degradation of DAAO. M+2 and M+3 citrate decreased from 39% to 35% and from 3% to 2 % due to the effect of dilution, respectively. In a two hour time-scale, the heavy labeling patterns were not achieved. **(C)** The MID of a-KG represents that M+4 labeling fraction was increased from 1 to 4 %. **(D)** The MID of glutamate demonstrated the same labeling pattern, suggesting these two metabolites were quickly equilibrated. Mass isotopomer distributions (MIDs) of the TCA cycle metabolites after 2 and 24 hours of incubation with 0, 1, 3 and 5 mM D-alanine in media containing [U-${{}^{13}C}_{6}]\mathrm{glucose}$. MIDs were determined based on **(E)** citrate **(F)** a-KG **(G)** succinate **(H)** fumarate **(I)** malate **(J)** aspartate.


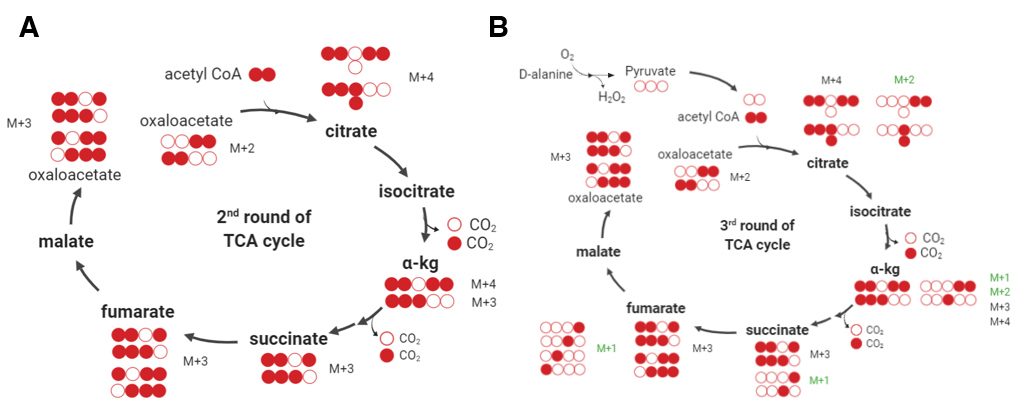


**Figure S6, related to Fig 5. (A)** Schematics representing the 2^nd^ round of TCA cycle. As M+2 labeled oxaloacetate enters the 2^nd^ round of TCA cycle, succinate, fumarate, and oxaloacetate can be M+3 at the end of the 2^nd^ round of TCA cycle. **(B)** Schematics representing the 3^rd^ round of TCA cycle with non-labeled pyruvate that can be introduced by the DAAO system. The green labeled mass isotopomer represents an artificial labeling pattern introduced by the non-labeled pyruvate. The black label represents the theoretical labeling pattern by [U-${{}^{13}C}_{6}]\mathrm{glucose}$. With the non-labeled acetyl-CoA, remaining TCA cycle metabolites can be either M+1 or M+2 instead of M+3 or higher.


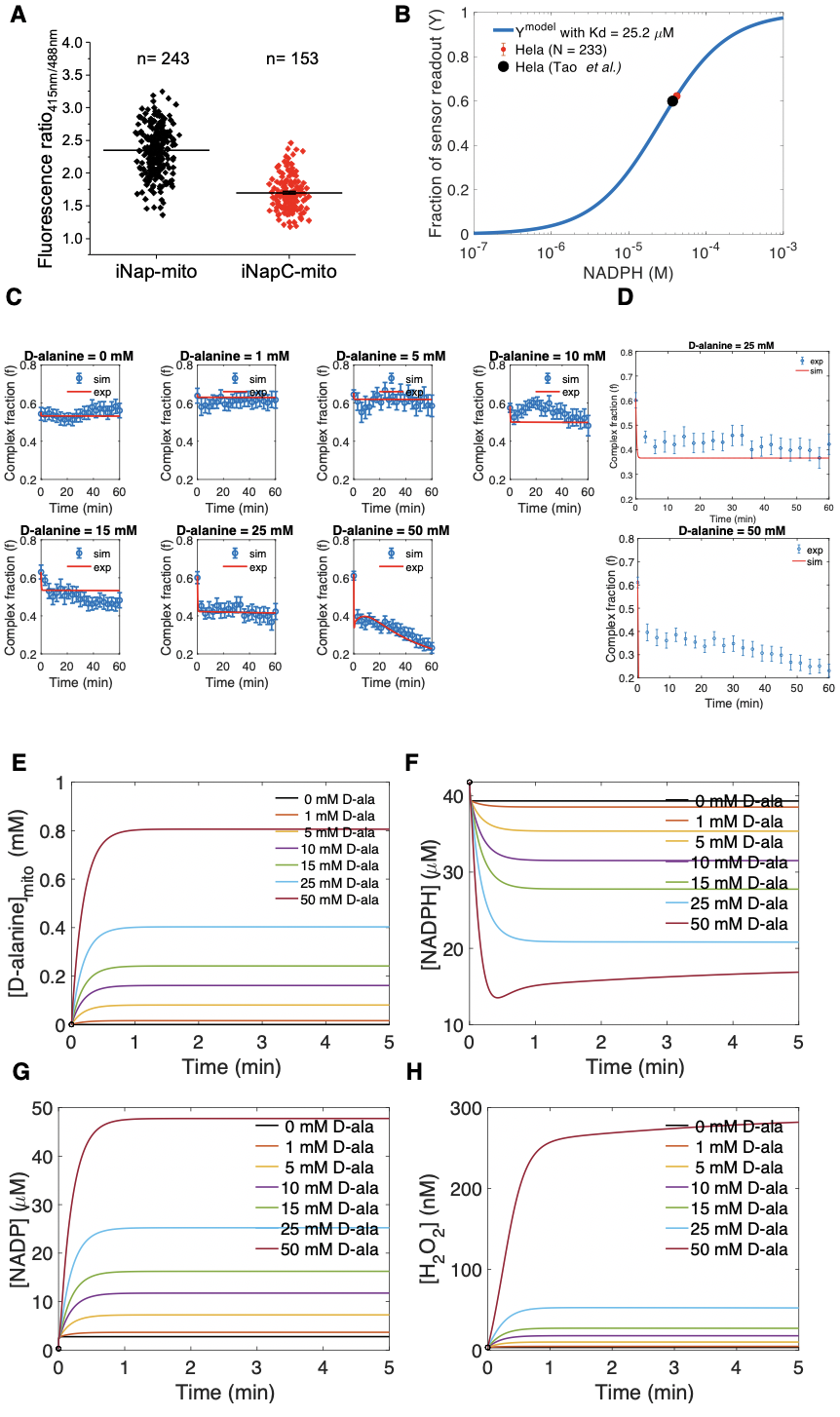


**Figure S7, related to Fig 6. (A)** The scatter plot represents fluorescence signals from iNap-mito and iNapC-mito with its mean and S.E.M. The mean of iNap-mito is 2.35 and that of iNapC-mito is 1.71. The normalized average signal is 1.38, which is equal to 62% of sensor being in a bound state with the NADPH after calibrating with the maximum and minimum signals. **(B)** The fraction was converted to concentration by equating $Y^{\mathrm{model}}$ and $Y^{\exp}$. Based on Tao paper, the NADPH level was determined 37 µM and its sensor occupancy were approximately 60% in this model. **(C)** The presence and absence of stress-dependent NADPH flux term**.** The model with fitted parameter values was run with different initial inputs and compared to the experimental data. The rate of NADPH included the $\alpha\times v_{H_{2}O_{2}}^{tot}$ term, which prevented a collapse of readout under 25 and 50 mM of D-alanine input. **(D)** The model was simulated with the stress-dependent NADPH flux term at 25 and 50 mM D-alanine conditions. The complex fraction (Y) decreased immediately within one minute. The error bar of experimental data represented the S.E.M. **(E)** The model simulation with the fitted parameter values. The model was run with different concentration of D-alanine and the concentration of intracellular D-alanine in mitochondria were predicted over the course of 5 minutes. **(F)** The model simulation for NADPH over the first 5 minutes. A rise of concentration under 50 mM D-alanine condition was due to the increased stress-dependent NADPH flux, preventing a continuous drop of NADPH level. **(G)** NADP level was tracked over 5 minutes. **(H)** The intracellular $H_{2}O_{2}$ level was traced over 5 minutes.


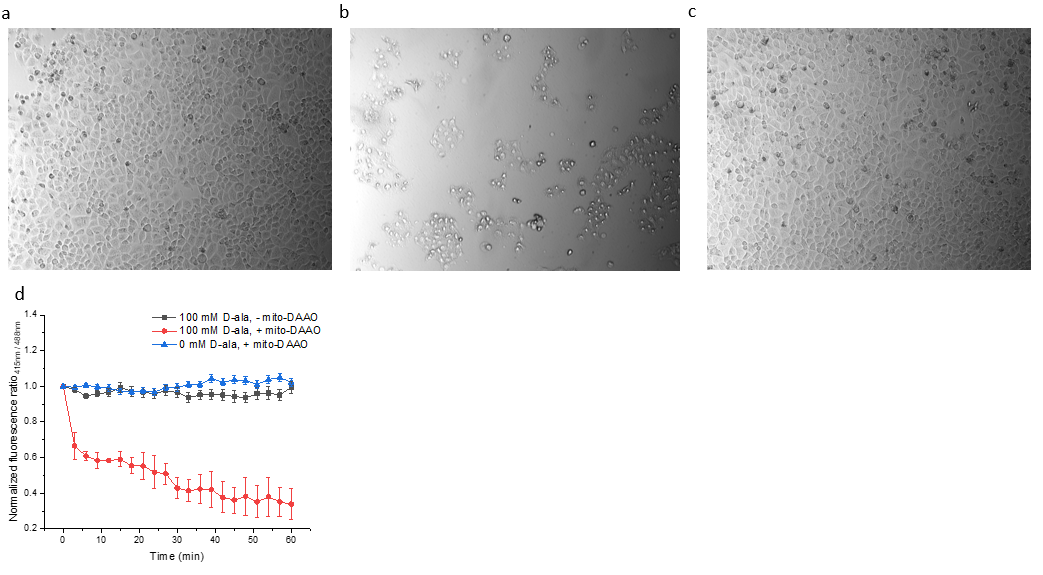


**Figure S8.** Transmitted images were taken after 24 hours of treatment with D-alanine of (a) 0 mM in HeLa-mito-DAAO, (b) 100 mM in HeLa-mito-DAAO, and (c) 100 mM in HeLa only. (D) represents the corresponding normalized fluorescence ratios of iNap-mito sensors that were recorded under conditions that represented in figure (a) - (c). Emission ratio values represent mean values of individual cells from at least two independent experiments $\pm SEM$. (n = 8, 12, 23, from experiments with 100 mM of D-alanine in HeLa, 100 mM in HeLa-mito-DAAO, and 0 mM in HeLa-mito-DAAO).

**Figure S9.** Relative pathway strengths between the pentose phosphate pathway (PPP) and glycolytic pathway. Hela cells that express either DAAO-mito or DAAO in $\left[ 1,2\text{-}{}^{13}{C_{2}} \right]\mathrm{glucose}$ isotope tracer media were stimulated with varying concentrations of D-alanine metabolites were collected at 0.5, 1, and 2 hrs.

**Table S1, related to Fig 1 and 6.** Parameters for the mitochondrial redox reactions.

|  | *Reaction* | *Parameter* | *Reference* |
| --- | --- | --- | --- |
| $r_{1}:$ | Intracellular $H_{2}O_{2}$ generation | $k_{gen,intraH_{2}O_{2}}=4.0\times{10}^{-6}M/s$ | ^49,50^ |
| $r_{2}^{*}:$ | $P\times\frac{A_{m}}{V_{\mathrm{cell}}}\times\left( \left[ D\text{-}\mathrm{alanine} \right]_{\mathrm{ex}} \right)\times V_{\mathrm{well}}$ | $P=6.92\times{10}^{\begin{aligned} -10 \\ \end{aligned}} cm/s$ | Fitted |
| $r_{3}^{*}:$ | $P\times A \times\left( \left[ D\text{-}\mathrm{alanine} \right]_{\mathrm{in}} \right)\times cell number$ |  |  |
| $r_{3}:$ | $k_{gen,H_{2}O_{2}}(\left[ D\text{-}\mathrm{alanine} \right]_{\mathrm{in}})$ | $k_{gen,H_{2}O_{2}}=8.83\times{10}^{-2}s^{-1}$ | Fitted |
| $r_{4}:$ | $k_{gen,NADPH}(\left[ \mathrm{NADP}^{+} \right])$ | $k_{gen,NADPH}=1.59 s^{-1}$ | Fitted |
| $r_{5}:$ | $k_{import,GSH}$ | $k_{import,GSH}=4.8\times{10}^{-7}M/s$ | ^82^ |
| $r_{6}:$ | $k_{degradation,GSH}$ | $k_{degradation,GSH}=3.2\times{10}^{-8}M/s$ | ^82^ |
| $v_{11}:$ | $k_{11}([GSSG])(\left[ \mathrm{NADPH} \right])$ | $k_{11}=3.2\times{10}^{6} {M^{-1}s}^{-1}$ | ^51^ |
| $v_{12}:$ | $k_{12}([GrxSSG])(\left[ \mathrm{GSH} \right])$ | $k_{12}=3.7\times{10}^{4} {M^{-1}s}^{-1}$ | ^52^ |
| $v_{13}:$ | $k_{13}\left( \left[ \mathrm{GrxSH} \right] \right)\left( \left[ \mathrm{PrSSG} \right] \right)$ | $k_{13}=1.2\times{10}^{4} {M^{-1}s}^{-1}$ | ^53^ |
| $v_{14}:$ | $k_{14}(\left[ \mathrm{PrSOH} \right])([GSH])$ | $k_{14}=1.2\times{10}^{5} {M^{-1}s}^{-1}$ | ^54,55^ |
| $v_{15}:$ | $k_{15}(\left[ \mathrm{PrSH} \right])([H_{2}O_{2}])$ | $k_{15}=1\times{10}^{2} {M^{-1}s}^{-1}$ | ^36^ |
| $v_{16}:$ | $k_{16}([GSH])$ | $k_{16}=7.4\times{10}^{-5} s^{-1}$ | ^34^ |
| $v_{21}:$ | $k_{21}(\left[ \mathrm{TrxSS} \right])(\left[ \mathrm{NADPH} \right])$ | $k_{21}=2\times{10}^{7} {M^{-1}s}^{-1}$ | ^16^ |
| $v_{22}:$ | $k_{22}(\left[ \mathrm{PrxSS} \right])([TrxSH])$ | $k_{22}=2.2\times{10}^{5} {M^{-1}s}^{-1}$ | ^56^ |
| $v_{23}:$ | $k_{23}(\left[ H_{2}O_{2} \right])([PrxSH2])$ | $k_{23}=2\times{10}^{7} {M^{-1}s}^{-1}$ | ^16^ |
| $v_{24}:$ | $k_{24}(\left[ H_{2}O_{2} \right])([PrxSOH])$ | $k_{24}=1.4\times{10}^{4} {M^{-1}s}^{-1}$ | ^36^ |
| $v_{25}:$ | $k_{25}([PrxSOOH])$ | $k_{25}=3\times{10}^{-3} s^{-1}$ | ^57^ |
| $v_{26}:$ | $k_{26}([PrxSOH])$ | $k_{26}=20s^{-1}$ | ^58^ |
| $v_{31}:$ | $k_{31}(\left[ \mathrm{TrxSH} \right])(\left[ \mathrm{PrSS} \right])$ | $k_{31}=1\times{10}^{2} {M^{-1}s}^{-1}$ | ^36^ |
| $v_{32}:$ | $k_{32}(\left[ H_{2}O_{2} \right])([PrSH2])$ | $k_{32}=1\times{10}^{2} {M^{-1}s}^{-1}$ | ^36^ |
| $v_{41}:$ | $k_{41}(\left[ H_{2}O_{2} \right])([GPxRd])$ | $k_{41}=6\times{10}^{7} {M^{-1}s}^{-1}$ | ^59^ |
| $v_{42}:$ | $k_{42}(\left[ \mathrm{GSH} \right])([GPxOx])$ | $k_{42}=4\times{10}^{4} {M^{-1}s}^{-1}$ | ^59^ |
| $v_{43}:$ | $k_{43}([GPxSSG])(\left[ \mathrm{GSH} \right])$ | $k_{43}=1\times{10}^{7} {M^{-1}s}^{-1}$ | ^59^ |
| $v_{51}:$ | $k_{51}(\left[ H_{2}O_{2} \right])([Prx5SH2])$ | $k_{51}=3\times{10}^{5} {M^{-1}s}^{-1}$ | ^83^ |
| $v_{52}:$ | $k_{52}([Prx5SOH])$ | $k_{52}=14.7 s^{-1}$ | ^83^ |
| $v_{53}:$ | $k_{53}(\left[ Prx5SS \right])([TrxSH])$ | $k_{53}=2\times{10}^{6} {M^{-1}s}^{-1}$ | ^83^ |
| $v_{61}:$ | $k_{61}(\left[ H_{2}O_{2} \right])([GPx4Rd])$ | $k_{61}=4.8\times{10}^{4} {M^{-1}s}^{-1}$ | ^84^ |
| $v_{62}:$ | $k_{62}(\left[ \mathrm{GSH} \right])([GPx4Ox])$ | $k_{62}=2\times{10}^{4} {M^{-1}s}^{-1}$ | ^84^ |
| $v_{71}:$ | Srx import | $k_{71}=1.23\times{10}^{-5} M/s$ | ^85^ |
| $r_{6}$ | $\alpha\times v_{H_{2}O_{2}}^{tot}$  $v_{H_{2}O_{2}}^{tot}=r_{1}+r_{3}-v_{15}-v_{23}-v_{24}-v_{32}-v_{41}$ | $\alpha=80.9$ | Fitted |

* $A_{m}=1.26\times{10}^{-9}m^{2},r_{2}^{*}\mathrm{and}r_{3}^{*} are units of mol per s$; cell = $7\times{10}^{5}$; volWell = $2\times{10}^{-6} m^{3}$; volCell = $4.19\times{10}^{-15} m^{3}$

**Table S2, related to Fig 1 and 6.** Initial concentrations of the mitochondrial redox model for Hela cells.

| Species | Initial concentration (M) | Reference |
| --- | --- | --- |
| $\left[ D\text{-}\mathrm{alanine} \right]_{\mathrm{ex}}$ | $0 to 50\times{10}^{-3}$ | Assigned |
| $\left[ D\text{-}\mathrm{alanine} \right]_{\mathrm{en}}$ | 0 | Assigned |
| ${[H}_{2}O_{2}]$ | $3.33\times{10}^{-9}$ | Calculated |
| $[Prx\text{-}{(SH)}_{2}]$ | $6\times{10}^{-5}$ | ^16^ |
| $[Prx\text{-}\mathrm{SS}]$ | $2.36\times{10}^{-7}$ | Calculated |
| $\left[ \mathrm{Trx}\text{-(}\mathrm{SH} \right)_{2}]$ | $7.7\times{10}^{-6}$ | ^60^ |
| $[Trx\text{-}\mathrm{SS}]$ | $7.54\times{10}^{-8}$ | ^60^ |
| $[GSSG]$ | $1.78\times{10}^{-6}$ | ^34^ |
| $[GSH]$ | $5\times{10}^{-3}$ | ^34^ |
| $[Grx\text{-}\mathrm{SSG}]$ | $1.8\times{10}^{-18}$ | Calculated |
| $[Grx\text{-}\mathrm{SH}]$ | $1\times{10}^{-6}$ | ^36^ |
| $[Pr\text{-}\mathrm{SSG}]$ | $2.78\times{10}^{-14}$ | Calculated |
| $[Pr\text{-}\mathrm{SH}]$ | $1\times{10}^{-9}$ | ^36^ |
| $[Pr\text{-}\mathrm{SOH}]$ | $5.56\times{10}^{-19}$ | Calculated |
| $[Pr\text{-}\mathrm{SS}]$ | $4.72\times{10}^{-7}$ | Calculated |
| $\left[ \Pr\text{-(}\mathrm{SH} \right)_{2}]$ | $1.09\times{10}^{-3}$ | ^34^ |
| $[GPx\text{-}\mathrm{Ox}]$ | $1.5\times{10}^{-11}$ | Calculated |
| $[GPx\text{-}\mathrm{SH}]$ | $1.5\times{10}^{-8}$ | ^16^ |
| $[GPx\text{-}\mathrm{SSG}]$ | $6.00\times{10}^{-14}$ | Calculated |
| $[Prx\text{-}\mathrm{SOH}]$ | $2\times{10}^{-7}$ | Calculated |
| $[Prx\text{-}\mathrm{SOOH}]$ | $2.67\times{10}^{-9}$ | Calculated |
| $[\mathrm{NADP}^{+}]$ | $4.18\times{10}^{-6}$ | Assigned |
| $[NADPH]$ | $4.18\times{10}^{-5}$ | Calculated |
| $[Prx5\text{-}{(SH)}_{2}]$ | $1.4\times{10}^{-5}$ | ^60^ |
| $[Prx5\text{-}\mathrm{SOH}]$ | $9.43\times{10}^{-10}$ | Calculated |
| $[Prx5\text{-}\mathrm{SS}]$ | $9.00\times{10}^{-10}$ | Calculated |
| $[GPx4\text{-}\mathrm{SH}]$ | $2.30\times{10}^{-7}$ | ^60^ |
| $[GPx4\text{-}\mathrm{Ox}]$ | $3.60\times{10}^{-13}$ | Calculated |
| $[GPx4\text{-}\mathrm{SSG}]$ | $7.29\times{10}^{-16}$ | Calculated |
| $[Srx]$ | $8.78\times{10}^{-9}$ | ^60^ |

**Table S3, related to Fig 1 and 6. Results of the sensitivity analysis for NADPH at time = 3 min**. The top 5 most sensitive parameters were represented at 0, 1, 5, 10, 15, 25, and 50 mM D-alanine perturbation. The stress-dependent transport coefficient became more sensitive as the perturbation increased.

| # | **0 mM** |  | **1 mM** |  | **5 mM** |  | **10 mM** |  |
| --- | --- | --- | --- | --- | --- | --- | --- | --- |
| 1 | $k_{gen,NADPH}$ | 0.064 | $k_{gen,NADPH}$ | 0.086 | $k_{gen,NADPH}$ | 0.186 | $k_{gen,NADPH}$ | 0.338 |
| 2 | $k_{gen,H_{2}O_{2},intra}$ | 0.057 | $k_{gen,H_{2}O_{2},intra}$ | 0.058 | $k_{Dala,trans}$ | 0.110 | $k_{Dala,trans}$ | 0.240 |
| 3 | $k_{16}$ | 0.006 | $k_{Dala,trans}$ | 0.021 | $k_{gen,H_{2}O_{2},intra}$ | 0.062 | $k_{gen,H_{2}O_{2},intra}$ | 0.068 |
| 4 | $k_{23}$ | 0.006 | $k_{23}$ | 0.009 | $k_{23}$ | 0.020 | $k_{23}$ | 0.041 |
| 5 | $k_{22}$ | 0.000 | $k_{16}$ | 0.006 | α | 0.015 | α | 0.037 |

| # | **15 mM** |  | **25 mM** |  | **50 mM** |  |
| --- | --- | --- | --- | --- | --- | --- |
| 1 | $k_{gen,NADPH}$ | 0.529 | $k_{gen,NADPH}$ | 1.093 | $k_{Dala,trans}$ | 6.290 |
| 2 | $k_{Dala,trans}$ | 0.389 | $k_{Dala,trans}$ | 0.731 | $k_{22}$ | 2.844 |
| 3 | $k_{gen,H_{2}O_{2},intra}$ | 0.075 | α | 0.188 | $k_{gen,NADPH}$ | 2.403 |
| 4 | $k_{23}$ | 0.070 | $k_{23}$ | 0.171 | α | 1.181 |
| 5 | α | 0.069 | $k_{22}$ | 0.106 | $k_{23}$ | 1.121 |

Rate constants appeared in the table represents $k_{11}\left( [GSSG] \right)\left( [NADPH] \right);k_{16}\left( \mathrm{GSH} \right); k_{21}\left( \left[ \mathrm{TrxSS} \right] \right)\left( \left[ \mathrm{NADPH} \right] \right); {k_{22}\left( \left[ \mathrm{PrxSS} \right] \right)\left( \left[ \mathrm{TrxSH} \right] \right); k}_{23}(\left[ H_{2}O_{2} \right])([PrxSH2])$
